# Recent Trends in Stratospheric Chlorine From Very Short‐Lived Substances

**DOI:** 10.1029/2018JD029400

**Published:** 2019-02-16

**Authors:** Ryan Hossaini, Elliot Atlas, Sandip S. Dhomse, Martyn P. Chipperfield, Peter F. Bernath, Anton M. Fernando, Jens Mühle, Amber A. Leeson, Stephen A. Montzka, Wuhu Feng, Jeremy J. Harrison, Paul Krummel, Martin K. Vollmer, Stefan Reimann, Simon O'Doherty, Dickon Young, Michela Maione, Jgor Arduini, Chris R. Lunder

**Affiliations:** ^1^ Lancaster Environment Centre Lancaster University Lancaster UK; ^2^ Rosenstiel School of Marine and Atmospheric Science (RSMAS) University of Miami Coral Gables FL USA; ^3^ School of Earth and Environment University of Leeds Leeds UK; ^4^ Department of Chemistry and Biochemistry Old Dominion University Norfolk VA USA; ^5^ Department of Chemistry University of Waterloo Waterloo ON Canada; ^6^ Department of Physics Old Dominion University Norfolk VA USA; ^7^ Scripps Institution of Oceanography University of California San Diego La Jolla CA USA; ^8^ National Oceanic and Atmospheric Administration (NOAA) Boulder CO USA; ^9^ NCAS University of Leeds Leeds UK; ^10^ Department of Physics and Astronomy University of Leicester Leicester UK; ^11^ National Centre for Earth Observation University of Leicester Leicester UK; ^12^ Climate Science Centre CSIRO Oceans and Atmosphere Aspendale Victoria Australia; ^13^ Laboratory for Air Pollution and Environmental Technology Empa, Swiss Federal Laboratories for Materials Science and Technology Duebendorf Switzerland; ^14^ School of Chemistry University of Bristol Bristol UK; ^15^ Department of Pure and Applied Sciences University of Urbino Urbino Italy; ^16^ Norwegian Institute for Air Research Kjeller Norway

**Keywords:** chlorine, stratosphere, VSLS, chloroform, dichloromethane, ozone

## Abstract

Very short‐lived substances (VSLS), including dichloromethane (CH_2_Cl_2_), chloroform (CHCl_3_), perchloroethylene (C_2_Cl_4_), and 1,2‐dichloroethane (C_2_H_4_Cl_2_), are a stratospheric chlorine source and therefore contribute to ozone depletion. We quantify stratospheric chlorine trends from these VSLS (VSLCl_tot_) using a chemical transport model and atmospheric measurements, including novel high‐altitude aircraft data from the NASA VIRGAS (2015) and POSIDON (2016) missions. We estimate VSLCl_tot_ increased from 69 (±14) parts per trillion (ppt) Cl in 2000 to 111 (±22) ppt Cl in 2017, with >80% delivered to the stratosphere through source gas injection, and the remainder from product gases. The modeled evolution of chlorine source gas injection agrees well with historical aircraft data, which corroborate reported surface CH_2_Cl_2_ increases since the mid‐2000s. The relative contribution of VSLS to total stratospheric chlorine increased from ~2% in 2000 to ~3.4% in 2017, reflecting both VSLS growth and decreases in long‐lived halocarbons. We derive a mean VSLCl_tot_ growth rate of 3.8 (±0.3) ppt Cl/year between 2004 and 2017, though year‐to‐year growth rates are variable and were small or negative in the period 2015–2017. Whether this is a transient effect, or longer‐term stabilization, requires monitoring. In the upper stratosphere, the modeled rate of HCl decline (2004–2017) is −5.2% per decade with VSLS included, in good agreement to ACE satellite data (−4.8% per decade), and 15% slower than a model simulation without VSLS. Thus, VSLS have offset a portion of stratospheric chlorine reductions since the mid‐2000s.

## Key Points

- Stratospheric chlorine from very short‐lived substances increased by 3.8 ppt/year over 2004–2017, with a growth slowdown in 2015–2017
- Chlorine from short‐lived substances improves model representation of upper stratospheric HCl trends
- Short‐lived chlorine offsets the 2004–2017 rate of upper stratospheric HCl decline by 15%

## 1 Introduction

The depletion of the global ozone layer by chlorine and bromine compounds is a well‐established and persistent environmental issue (e.g., Solomon, 1999). It is predominately caused by chlorine and bromine radicals released from long‐lived halocarbons, such as chlorofluorocarbons (CFCs) and halons, whose production is now controlled by the UN Montreal Protocol and its amendments (e.g., WMO, 2014). Owing to the Protocol's ongoing success, the atmospheric abundances of total chlorine and bromine are declining (e.g., Carpenter et al., 2014; Froidevaux et al., 2006; Montzka et al., 2003) and the ozone layer is projected to return to pre‐1980s levels in the middle to latter half of this century in consequence (e.g., Chipperfield et al., 2017; Dhomse et al., 2018; Eyring et al., 2010; Solomon et al., 2016).

While the science underpinning ozone depletion is well understood, uncertainties in emissions of certain ozone‐depleting compounds exist, which have the potential to influence future ozone projections. Notably, for example, long‐term surface measurements show that emissions of CFC‐11 have likely increased since 2012, despite its reported production being near zero (Montzka et al., 2018). Another example is the recent indication of increasing dichloromethane emissions since the early 2000s, based on a network of surface observations (Hossaini et al., 2015a; Hossaini et al., 2017) and measurements made in the upper troposphere (Leedham Elvidge et al., 2015; Oram et al., 2017). Dichloromethane (CH_2_Cl_2_), along with chloroform (CHCl_3_), perchloroethylene (C_2_Cl_4_), and 1,2‐dichloroethane (C_2_H_4_Cl_2_), among others, are so‐called Very Short‐Lived Substances (VSLS)—compounds with mean surface lifetimes typically less than 6 months (e.g., Ko et al., 2003).

Over the past decade it has become clear that (a) natural brominated VSLS (e.g., CHBr_3_) are a significant source of stratospheric bromine (e.g., Sala et al., 2014; Salawitch et al., 2005; Sturges et al., 2000; Wales et al., 2018), and (b) chlorinated VSLS (Cl‐VSLS) are a small, but potentially increasing, source of stratospheric chlorine (Hossaini et al., 2015b). CH_2_Cl_2_ is the most abundant Cl‐VSLS and its tropospheric abundance has increased approximately twofold since the early 2000s. In contrast, CHCl_3_ has remained relatively stable since the mid‐1990s, while C_2_Cl_4_ has been in long‐term decline over the same period (Carpenter et al., 2014). The above Cl‐VSLS are expected to have significant or predominately anthropogenic sources (Montzka et al., 2011). For example, CH_2_Cl_2_ is widely used as a solvent and in the production of foam agents, among other applications (Feng et al., 2018). A substantial portion of the estimated present‐day global CH_2_Cl_2_ emission rate of ~1 Tg year (Hossaini et al., 2017) is expected to occur in Asia (Oram et al., 2017). While no long‐term C_2_H_4_Cl_2_ surface record exists, observed interhemispheric gradients suggest that it too has anthropogenic sources (Hossaini et al., 2016a).

Carpenter et al. (2014) estimated that Cl‐VSLS contributed ~95 (50–145) parts per trillion (ppt) Cl to the stratospheric loading of inorganic chlorine in 2012. Hossaini et al. (2015b) used a global model to investigate the trend in stratospheric chlorine from Cl‐VSLS between 2005 and 2013. They estimated a mean increase of 3.7 ppt Cl/year over this period, with the positive trend reflecting increasing CH_2_Cl_2_. Although the impact of this additional chlorine on stratospheric ozone is expected to have been modest over the last decade (Chipperfield et al., 2018), possible future increases in Cl‐VSLS emissions could influence the time scale for stratospheric ozone recovery, particularly in polar regions (Hossaini et al., 2017). Such projections carry large uncertainties; however, they are important to consider to provide bounds on potential resulting ozone changes, especially given that large CH_2_Cl_2_ emission increases from major economies are projected under *business as usual* scenarios until 2030 (Feng et al., 2018). On the same basis, it is important that historical trends in stratospheric chlorine from VSLS are well quantified, so that possible future changes can be gauged from an accurate baseline.

The aim of this paper is to provide an up‐to‐date assessment of the stratospheric chlorine loading due to Cl‐VSLS (CH_2_Cl_2_, CHCl_3_, C_2_Cl_4_, C_2_H_4_Cl_2_, and C_2_HCl_3_) and to examine trends over the 2000–2017 period. To achieve this, we use a 3‐D chemical transport model (CTM) supported by a range of atmospheric observations. This includes novel high‐altitude aircraft measurements of these compounds from campaigns in 2015 and 2016. Our analysis also focusses on (a) whether a signature of historical Cl‐VSLS trends is present in long‐term stratospheric HCl records, (b) quantifying the sensitivity of stratospheric chlorine from VSLS to uncertainties in VSLS chemistry and removal processes, and (c) estimating the contribution from other less prominent Cl‐VSLS, based on available observational data. Section 2 describes the CTM and the model experiments that were performed. Section 3 describes the various observational data sets. Section 4 presents our results, and conclusions are given in section 5.

## 2 Model and Experiments

### 2.1 CTM

TOMCAT/SLIMCAT is an offline 3‐D CTM (Chipperfield, 2006; Monks et al., 2017) that has been widely used for studies of chemistry and transport in the troposphere (e.g., McNorton et al., 2016; Richards et al., 2013) and stratosphere (e.g., Chipperfield, 2006; Dhomse et al., 2016, 2015). The CTM is forced by meteorological fields (winds, temperature, and humidity) taken from the European Centre for Medium‐Range Weather Forecasts ERA‐Interim reanalysis (Dee et al., 2011). Tracer advection is based on a conservation of second‐order moments scheme (Prather, 1986) and convective transport is parameterized based on the mass flux scheme of Tiedtke (1989), as described by Feng et al. (2011). Turbulent boundary layer mixing follows the nonlocal scheme of Holtslag and Boville (1993). The configuration of the model used here employs a hybrid sigma‐pressure (*σ‐p*) vertical coordinate with 60 verticals levels extending from the surface to ~60 km. Simulations were performed at a horizontal resolution of 2.8° × 2.8°.

The tropospheric model configuration described above has been used extensively in VSLS‐related studies (e.g., Hossaini et al., 2016b) and employs a simplified offline chemistry scheme, whereby monthly mean fields of the hydroxyl radical (OH) concentration are prescribed based on the climatology produced for the TransCom‐CH_4_ project (Patra et al., 2011). We assume a fixed tropospheric Cl atom concentration, [Cl] = 1.3 × 10^3^ atoms per cubic centimeter, based on the tropospheric mean [Cl] calculated by a recent global model study (Hossaini et al., 2016a). Note, this configuration of the model is here applied to calculate the stratospheric input of Cl‐VSLS and their products and does not contain detailed stratospheric chemistry. A stratospheric model configuration, with a very detailed stratospheric chemistry scheme, is later used to investigate HCl trends (see section 4.5) with and without Cl‐VSLS.

### 2.2 Source Gas Boundary Conditions and Chemistry

Four major Cl‐VSLS were considered in our control (“BASE”) simulation (CH_2_Cl_2_, CHCl_3_, C_2_Cl_4_, and C_2_H_4_Cl_2_). Constraint on the surface abundance of these source gases (SGs) was applied using a latitude‐dependent boundary condition based on observed mole fractions (SG_MF_), varying in five bands (60–90°N, 30–60°N, 0–30°N, 0–30°S, and 30–90°S). Although simple, this approach has been shown to reproduce upper tropospheric observations and trends of these compounds well (Hossaini et al., 2015b). For CH_2_Cl_2_ and C_2_Cl_4_, SG_MF_ is based on NOAA surface measurements and varies annually (e.g., Montzka et al., 2018). For CHCl_3_, SG_MF_ is based on AGAGE measurements (e.g., Prinn et al., 2000) and also varies annually (Text S1 and Figure S1). For C_2_H_4_Cl_2_ and C_2_HCl_3_ (a very minor compound), no long‐term surface records are available, thus SG_MF_ is estimated based on observations from the 2009–2012 HIPPO mission (e.g., Wofsy et al., 2011, 2012), with no time trend in SG_MF_ applied. In addition to VSLS, long‐lived carbon tetrachloride (CCl_4_) and methyl chloroform (CH_3_CCl_3_) are included in the model. Like VSLS, these compounds are a source of phosgene, which constitutes a significant component of stratospheric chlorine (e.g., Fu et al., 2007). For CCl_4_ and CH_3_CCl_3_, a global SG_MF_ was prescribed.

For each VSLS, the BASE run considered chemical loss through OH oxidation, Cl atom oxidation, and photolysis, with the relevant kinetic parameters (rate constants, absorption cross sections, see Tables S1 and S2) mostly taken from the Jet Propulsion Laboratory data evaluation (Burkholder et al., 2015). Sensitivity experiments were performed to assess the significance of tropospheric VSLS oxidation by Cl (see section 2.4).

### 2.3 Product Gases

The oxidation of chlorine‐containing VSLS has been considered in past WMO/UNEP Ozone Assessments (e.g., Ko et al., 2003) and in recent modeling work (Hossaini et al., 2016a). Here we adopted a simplified treatment of product gas (PG) chemistry, considering two idealized PG tracers, phosgene (COCl_2_) and inorganic chlorine (Cl_y_), thus allowing many long integrations of the model to be performed.

#### 2.3.1 Phosgene

COCl_2_ is an expected major product of CHCl_3_ and C_2_Cl_4_ oxidation (Ko et al., 2003), as demonstrated by experimental and theoretical studies (e.g., Christiansen & Francisco, 2010a, 2010b; Tuazon et al., 1988). The COCl_2_ yield from reaction of CHCl_3_, C_2_Cl_4_, and C_2_HCl_3_ with OH has been reported to be 1.0, 0.47, and 0.4, respectively (Kindler et al., 1995). For these compounds, COCl_2_ production in the model is calculated based on the above experimentally determined yields.

Little is currently known regarding the yield of COCl_2_ from CH_2_Cl_2_ oxidation, with limited information in the literature. Although some experimental evidence for COCl_2_ production from CH_2_Cl_2_ oxidation is available (Catoire et al., 1996; Sanhueza & Heicklen, 1975; Spence et al., 1976), COCl_2_ was not noted as a major product of CH_2_Cl_2_ by Ko et al. (2003). In this work, we calculate COCl_2_ production from CH_2_Cl_2_ “online” in the model based on the principal steps involved in the oxidation chain, as outlined below.

The initial oxidation of CH_2_Cl_2_ by either OH or Cl atoms ((R1), (R2)) produces a chlorinated peroxy radical (CHCl_2_O_2_):

(R1) $${\mathrm{CH}}_{2}{\mathrm{Cl}}_{2}+\mathrm{OH}\;\left(+O_{2}\right)\to {\text{CHCl}}_{2}O_{2}+H_{2}O$$

(R2) $${\mathrm{CH}}_{2}{\mathrm{Cl}}_{2}+\mathrm{Cl}\;\left(+O_{2}\right)\to {\text{CHCl}}_{2}O_{2}+\mathrm{HCl}$$

The peroxy radical can undergo one of several different reactions. Under high NO_x_ conditions, such as in urban areas, reaction with NO (or NO_3_) will likely dominate loss of CHCl_2_O_2_, leading to production of the alkoxy radical, CHCl_2_O.

(R3) $${\text{CHCl}}_{2}O_{2}+\mathrm{NO}\to {\text{CHCl}}_{2}O+{\mathrm{NO}}_{2}$$

(R4) $${\text{CHCl}}_{2}O_{2}+{\mathrm{NO}}_{3}\to {\text{CHCl}}_{2}O+{\mathrm{NO}}_{2}+O_{2}$$

(R3) and (R4) are not phosgene forming channels as CHCl_2_O is expected to decompose to CHClO + Cl (e.g., Catoire et al., 1996). The atmospheric fate of CHClO is poorly known but sinks are expected to include atmospheric removal via heterogeneous and multiphase deposition processes (e.g., Ko et al., 2003; Toyota et al., 2004).

Alternatively, CH_2_ClO_2_ may react with HO_2_ or RO_2_ (e.g., Catoire et al., 1996), especially in low NO_x_ regions, through several phosgene‐forming channels (R5, R6).

(R5a) $${\text{CHCl}}_{2}O_{2}+{\mathrm{HO}}_{2}\;\to {\text{CHCl}}_{2}\mathrm{OOH}+O_{2},$$

(R5b) $$\to {\text{COCl}}_{2}+H_{2}O+O_{2}$$

(R5c) $$\to \text{CHClO}+\text{HOCl}+O_{2}$$

(R6a) $${\text{CHCl}}_{2}O_{2}+{\mathrm{CH}}_{3}O_{2}\;\to {\text{CHCl}}_{2}O+\text{products},$$

(R6b) $$\to {\text{CHCl}}_{2}\text{OH}+\text{products}$$

(R6c) $$\to {\text{COCl}}_{2}+\text{products}$$

Branching ratios for reactions with HO_2_ were taken from the most recent IUPAC evaluation (Atkinson et al., 2008), while for RO_2_ they are from the Master Chemical Mechanism version 3.3.1 (http://mcm.leeds.ac.uk/MCM/). For (R5a), (R5b), (R5c) the branching ratios are 0, 0.7 and 0.3, respectively, while for (R6a), (R6b), (R6c) they are 0.6, 0.2 and 0.2. Note, in addition to (R5b) and (R6c), COCl_2_ is also expected to be produced from (R6b), owing to the subsequent degradation of the CHCl_2_OH alcohol (Master Chemical Mechanism, v3.3.1).

Based on the above, the COCl_2_ yield (Y) from CH_2_Cl_2_ oxidation is computed in all model grid boxes on every time step according to equation (1), where k_3_, k_4_, k_5_, and k_6_ are rate constants for the reactions of CHCl_2_O_2_ with NO, NO_3_, HO_2_, and CH_3_O_2_, respectively.

(1) $$Y=\left(0.{\text{7k}}_{5}\left[{\mathrm{HO}}_{2}\right]+0.{\text{4k}}_{6}\left[{\mathrm{CH}}_{3}O_{2}\right]\right)/\left(k_{3}\left[\mathrm{NO}\right]+k_{4}\left[{\mathrm{NO}}_{3}\right]+k_{5}\left[{\mathrm{HO}}_{2}\right]+k_{6}\left[{\mathrm{CH}}_{3}O_{2}\right]\right).$$

An expression for COCl_2_ production from CH_2_Cl_2_ is thus: CH_2_Cl_2_ + OH → Y COCl_2_ + (2‐2Y) Cl_y_. Typical Y values strongly dependent on the local NO_x_ concentration and are found to be in the range of ~0.05 to 0.1 over industrialized regions and >0.3 over the oceans (see discussion in Text S2). Separate COCl_2_ tracers were included in the model, allowing its production from individual SGs to be tagged. In addition to production from VSLS, the model also considers COCl_2_ derived from long‐lived CCl_4_ and CH_3_CCl_3_, the former of which is the main stratospheric phosgene source (Kindler et al., 1995).

(R7) $${\mathrm{CCl}}_{4}+\mathrm{h\nu}\to {\text{COCl}}_{2}+\text{2Cl}$$

(R8) $${\mathrm{CH}}_{3}{\mathrm{CCl}}_{3}+\mathrm{OH}\to {\text{COCl}}_{2}+\mathrm{Cl}$$

Removal of COCl_2_ in the model occurs through photolysis (R9) and by washout. The latter is approximated in the model troposphere using a prescribed washout lifetime of 58 days (Kindler et al., 1995; Ko et al., 2003). The sensitivity of our results to this assumption and to uncertainties in the COCl_2_ yield calculation were examined (see also section 2.4).

(R9) $${\text{COCl}}_{2}+\mathrm{hv}\to \text{2Cl}+\mathrm{CO}$$

#### 2.3.2 Inorganic Chlorine (Cl_y_)

All non‐COCl_2_ PGs arising from CHCl_3_, CH_2_Cl_2_, C_2_Cl_4_, C_2_H_4_Cl_2_, and C_2_HCl_3_ oxidation were grouped in five generic Cl_y_ tracers, without further partitioning. These five Cl_y_ tracers were tagged allowing their production to be tracked to parent SGs. In the troposphere, Cl_y_ is dominated by highly soluble HCl (e.g., Hossaini et al., 2016a). The washout lifetime of Cl_y_ in the model was set to 5 days, based on the reported Cl_y_ lifetime from a recent detailed GEOS‐Chem 3‐D model study (Sherwen et al., 2016). The sensitivity of our findings to this lifetime were examined.

### 2.4 Base Simulation and Sensitivity Experiments

Our BASE (control) simulation, described in the previous sections, was spun‐up for a period of 5 years and then run over an 18‐year analysis period (2000 to 2017). Additionally, a series of model sensitivity runs were performed in which various parameters and processes were adjusted one‐by‐one with respect to the BASE run (Table 1). In EXP1, the CH_2_Cl_2_ and C_2_Cl_4_ surface boundary conditions were prescribed according to AGAGE data (compared to NOAA in the BASE run). In EXP2, the Cl atom sink of VSLS was switched off. In EXP3, tropospheric wet removal of COCl_2_ and Cl_y_ was switched off to give an upper limit to their potential contribution to stratospheric chlorine. In EXP4, repeating year 2000 meteorology was used throughout the simulation so that the influence of varying dynamics on the stratospheric input of VSLS could be assessed (i.e., comparing BASE to EXP4).

**Table 1:** Summary and Brief Description of TOMCAT/SLIMCAT Model Experiments

Model run	Description
BASE	Control run: see main text
EXP1	As BASE but CH_2_Cl_2_ and C_2_Cl_4_ surface boundary conditions from AGAGE
EXP2	As BASE but no Cl sink of source gases
EXP3	As BASE but upper limit of PGI (no tropospheric Cl_y_ and COCl_2_ removal)
EXP4	As BASE but repeating meteorology
EXP5	As BASE but 25% increase in tropospheric [OH]
EXP6	As BASE but 25% decrease in tropospheric [OH]
EXP7	As BASE but fixed phosgene yield (Y) from CH_2_Cl_2_ of 1
EXP8	As EXP3 but fixed phosgene yield (Y) from CH_2_Cl_2_ of 1

*Note*. PGI = Product Gas Injection.

In EXP5 and EXP6, tropospheric [OH] was increased or decreased by 25%, respectively. This OH perturbation was chosen as it is approximately equal to the spread in tropospheric [OH] from the models that took part in the ACCMIP intercomparison (Voulgarakis et al., 2013). The COCl_2_ yield from CH_2_Cl_2_ (i.e., Y in equation (1)) was fixed to 1 globally in EXP7 and EXP8, which were otherwise identical to the BASE run or EXP3, respectively. The results from these sensitivity runs are discussed in section 4.4, in the context of their influence on total chlorine from VSLS delivered to the stratosphere.

## 3 Observations

### 3.1 High‐Altitude Aircraft Measurements

Cl‐VSLS observations from eight NASA aircraft missions between 2004 and 2016 were considered in this work (Table 2). As the measurements were obtained by the same group (the University of Miami), the possible influence of different calibration scales (used by different laboratories) on reported mole fractions is minimized. Each of the aircraft campaigns measured the principal Cl‐VSLS, CH_2_Cl_2_, CHCl_3_, C_2_Cl_4_, and C_2_H_4_Cl_2_. Such observations are particularly valuable in the case of C_2_H_4_Cl_2_, due to the absence of long‐term surface measurements, but have yet to be examined in detail. In addition to the above compounds, measurements of other relatively minor Cl‐VSLS are available from some of the campaigns. These include chlorobenzene (C_6_H_5_Cl), chloroethane (C_2_H_5_Cl), and trichloroethylene (C_2_HCl_3_). Similarly, in the absence of long‐term surface measurements, the aircraft data provide useful constraint on their tropospheric abundance.

**Table 2:** Summary of Aircraft Campaign Data Used in This Study

Mission	Mon/year	Area	Species measured	Ref
Pre‐AVE	January–February 2004	Central America	CH_2_Cl_2_/CHCl_3_/C_2_Cl_4_/C_2_H_4_Cl_2_/C_2_HCl_3_	a, e
CR‐AVE	January–February 2006	Central America	CH_2_Cl_2_/CHCl_3_/C_2_Cl_4_/C_2_H_4_Cl_2_/C_2_H_5_Cl	a, f
TC4	August 2007	Central America	CH_2_Cl_2_/CHCl_3_/C_2_Cl_4_/C_2_H_4_Cl_2_/C_2_HCl_3_	b, g
ATTREX	November 2011	E. Pacific	CH_2_Cl_2_/CHCl_3_/C_2_Cl_4_/C_2_H_4_Cl_2_	c, d, h
ATTREX	February–March 2013	E. Pacific	CH_2_Cl_2_/CHCl_3_/C_2_Cl_4_/C_2_H_4_Cl_2_/C_6_H_5_Cl	
ATTREX	January–March 2014	W. Pacific	CH_2_Cl_2_/CHCl_3_/C_2_Cl_4_/C_2_H_4_Cl_2_/C_2_HCl_3_/C_6_H_5_Cl	
VIRGAS	October 2015	Gulf of Mexico	CH_2_Cl_2_/CHCl_3_/C_2_Cl_4_/C_2_H_4_Cl_2_/C_6_H_5_Cl	i
POSIDON	October 2016	W. Pacific	CH_2_Cl_2_/CHCl_3_/C_2_Cl_4_/C_2_H_4_Cl_2_/C_2_HCl_3_/C_6_H_5_Cl	j

a Hagan et al. (2004), Park et al. (2007).

b Pfister et al. (2010).

c Jensen et al. (2017).

d Navarro et al. (2015).

e https://espoarchive.nasa.gov/archive/browse/pre_ave

f https://espo.nasa.gov/ave‐costarica2/

g https://espo.nasa.gov/tc4/

h https://espo.nasa.gov/attrex/content/ATTREX

i https://www‐air.larc.nasa.gov/missions/virgas/

j https://espo.nasa.gov/posidon/

The location of the measurement campaigns is indicated in Figure S2. The campaigns sampled around Central America (Pre‐AVE 2004, CR‐AVE 2006, TC4 2007, ATTREX 2011, VIRGAS 2015), the East Pacific (ATTREX 2013), and the West Pacific (ATTREX 2014, POSIDON 2016). Measurements of Cl‐VSLS from the two most recent campaigns (VIRGAS and POSIDON) have yet to be reported in the literature. The VIRGAS (Volcano‐plume Investigation Readiness and Gas‐phase & Aerosol Sulfur) mission was conducted around the Gulf of Mexico in October 2015 (https://www.esrl.noaa.gov/csd/projects/virgas/). Measurements of Cl‐VSLS were obtained from whole air samples collected on board six flights of the NASA WB‐57 high‐altitude research aircraft. The POSIDON (Pacific Oxidants, Sulfur, Ice, Dehydration, & Convection) mission was centered around Guam in the tropical West Pacific (https://espo.nasa.gov/posidon/). Cl‐VSLS measurements were also obtained from whole air samples during 10 WB‐57 flights in October.

### 3.2 Satellite Measurements of COCl_2_ and HCl

We consider atmospheric measurements of COCl_2_ from the Atmospheric Chemistry Experiment Fourier Transform Spectrometer (ACE‐FTS), onboard the SCISAT satellite (Bernath, 2017). The ACE COCl_2_ data have been previously described in Fu et al. (2007) and Brown et al. (2011), and briefly considered by Hossaini et al. (2015b). Here we use version 3.5/3.6 ACE COCl_2_ data in 2014 and 2015 to assess the fidelity of the model COCl_2_ simulation in the tropical upper troposphere/lower stratosphere. For HCl, we consider the ACE measurement data set described by Bernath and Fernando (2018). Briefly, ACE HCl volume mixing ratios (version 3.5/3.6) were filtered at each pressure level by removing all values that were outside 2.5 standard deviations from the median. Quarterly averages were obtained for December–February, March–May, June–August, and September–November at each pressure level, making a HCl time series covering March–May 2004 to September–November 2017. The time series at each pressure level was deseasonalized and linear trends were calculated as described by Bernath and Fernando (2018). These trends are considered in section 4.5, with corresponding model trends calculated in the same fashion.

The standard error on the ACE and model HCl trend estimates was calculated using the method of Weatherhead et al. (1998), following Bernath and Fernando (2018), which includes a term considering the effect of first‐order autocorrelation in the time series of residuals. In section 4.5, trend errors are presented with ±2 standard error uncertainty.

## 4 Results and Discussion

### 4.1 SGs Lifetimes

**Figure 1:**
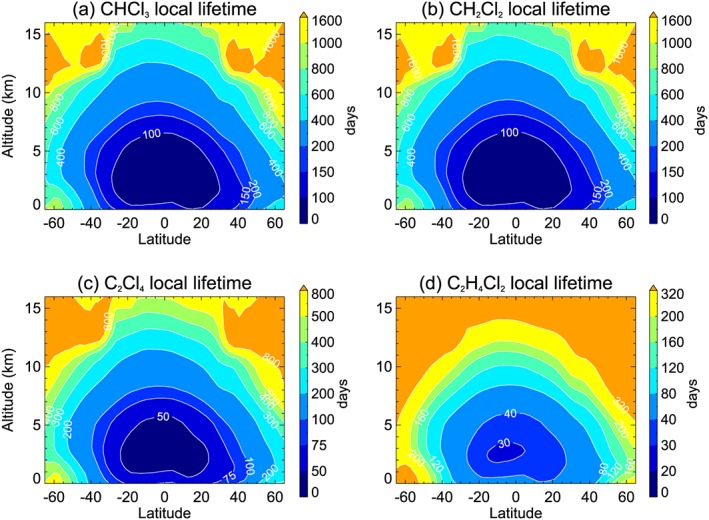
Local lifetime (days) of (a) CHCl_3_, (b) CH_2_Cl_2_, (c) C_2_Cl_4_ and (d) C_2_H_4_Cl_2_. The annual zonal mean local lifetime (τ_tot_) is calculated from partial lifetimes with respect to OH oxidation and photolysis (see section 4.1). Note the differing scales between panels.

We first consider the tropospheric lifetimes of CH_2_Cl_2_, CHCl_3_, C_2_Cl_4_, and C_2_H_4_Cl_2_. The total local lifetime (τ_tot_) of each SG is shown in Figure 1 (expressed as an annual and zonal mean). The total local lifetime of VSLS (e.g., Ko et al., 2003) is defined from the partial SG lifetimes with respect to oxidation via OH (τ_OH_) and photolysis (τ_hν_); that is, τ_tot_^−1^ = (τ_OH_)^−1^ + (τ_hν_)^−1^. The lifetime data presented in Figure 1 are based on EXP2; that is, the model run that considers these sinks but not oxidation of SGs via Cl atoms.

As with all VSLS, local lifetimes vary substantially with location (and season). For CHCl_3_ and CH_2_Cl_2_, the two SGs with the longest lifetimes, annual mean τ_tot_ for the tropical boundary layer is ~102 days (Table 3). Similarly, τ_tot_ is ~61 and ~43 days for C_2_Cl_4_ and C_2_H_4_Cl_2_, respectively. These calculated lifetimes are within ~10% of those reported by Carpenter et al. (2014). As has been reported for brominated VSLS, notably CH_2_Br_2_ (Hossaini et al., 2010), the local lifetime of chlorinated VSLS varies substantially with altitude. For example, in the cold tropical upper troposphere (~10 km), where temperature‐dependent loss rates via OH are relatively slow, τ_tot_ is a factor of around two to three times larger for each compound, compared to at the surface.

**Table 3:** Annual Mean Local Lifetime of Cl‐VSLS (Days) With Respect to OH Oxidation and Comparison to WMO (2014)

	Boundary layer		Upper troposphere (~10 km)	
Species	This work	WMO (2014)	This work	WMO (2014)
CHCl_3_	102 (93–114)	112 (100–136)	252 (245–258)	190 (182–197)
CH_2_Cl_2_	102 (93–114)	109 (98–133)	245 (238–250)	179 (171–185)
C_2_Cl_4_	61^a^ (55–68)	67 (60–81)	158^b^ (154–162)	119 (114–123)
C_2_H_4_Cl_2_	43 (39–48)	47 (42–58)	127 (124–130)	(86–93)

*Note*. The values in parentheses indicate seasonal range.

a 49 (45–53) days when C_2_Cl_4_ + Cl reaction included.

b 86 (84–87) days when C_2_Cl_4_ + Cl reaction included.

**Figure 2:**
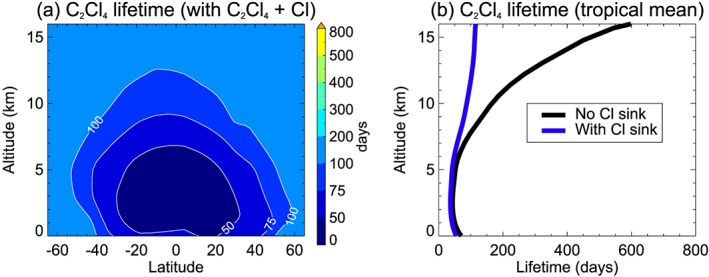
(a) C_2_Cl_4_ local lifetime (days) when the C_2_Cl_4_ + Cl sink is considered (in addition to loss via OH and photolysis). For comparison, the contour levels and scale are the same as those in panel (d) of Figure 1. (b) Tropical mean profile of C_2_Cl_4_ local lifetime calculated with (blue) and without (black) the C_2_Cl_4_ + Cl sink.

For CHCl_3_, CH_2_Cl_2_, and C_2_H_4_Cl_2_, Cl atoms are found to be a negligible tropospheric sink. As photolysis is also a negligible tropospheric sink (Carpenter et al., 2014), τ_tot_ effectively equals τ_OH_ for these species. However, for C_2_Cl_4_ we find that its local lifetime is strongly affected by inclusion of the tropospheric Cl sink (i.e., comparing EXP2 with the BASE run). For example, τ_tot_ varies from ~49 days in the tropical boundary layer to ~86 days at 10 km, when C_2_Cl_4_ + Cl is included (BASE simulation). Excluding the Cl sink (i.e., EXP2) increases these values to ~61 and ~158 days, respectively; that is, τ_tot_ is up to a factor ~2 larger (Table 3). Recall that EXP2 assumes a background tropospheric [Cl] of 1.3 × 10^3^ atoms per cubic centimeter, based on a recent global model estimate (Hossaini et al., 2016a). In section 4.2 we consider whether inclusion of the Cl atom sink improves model/measurement agreement of C_2_Cl_4_ in the upper troposphere, where its impact is greatest (Figure 2).

While the concept of a single, globally applicable, lifetime (defined by global burden/global removal rate) is generally not appropriate for VSLS, such values are used by box models to infer surface emissions (e.g., WMO, 2014). On this basis and for completeness, we report the following mean lifetimes from this study: 168 (CH_2_Cl_2_), 174 (CHCl_3_), 101 (C_2_Cl_4_, no Cl sink), 68 (C_2_Cl_4_, with Cl sink), and 77 days (C_2_H_4_Cl_2_).

### 4.2 SG Injection

**Figure 3:**
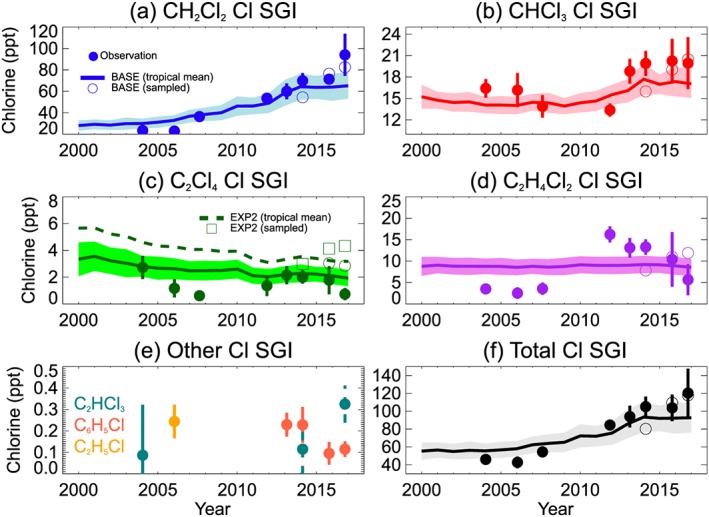
Stratospheric source gas chlorine injection (ppt Cl) from (a) CH_2_Cl_2_, (b) CHCl_3_, (c) C_2_Cl_4_, (d) C_2_H_4_Cl_2_, (e) other very short‐lived substances, and (f) total SGI. Model results (solid lines, shading denotes ±1σ) are from experiment BASE (tropical annual means) forced by surface data (section 2.2). Observed quantities (filled circles) are averages (vertical bars denote ±1 standard deviation) for available aircraft data between 16.5–17.5 km in the tropics (±20° latitude) from the following campaigns: 2004 (Pre‐AVE), 2006 (CR‐AVE), 2007 (TC4), 2011 (ATTREX), 2013 (ATTREX), 2014 (ATTREX), 2015 (VIRGAS), and 2016 (POSIDON). The location of these campaigns is summarized in Figure S2. The open symbols represent BASE output sampled at the measurement location/times. For C_2_Cl_4_ (panel c), model output from EXP2 (dashed line) is also shown; that is, not including the C_2_Cl_4_ + Cl sink. Note, “other very short‐lived substances” (C_2_HCl_3_, C_6_H_5_Cl, and C_2_H_5_Cl) that are observed but not modeled are not counted in total SGI (panel f) as their contribution seems insignificant. SGI = source gas injection.

We differentiate between chlorine input to the stratosphere from (a) chlorine atoms in the form of emitted SGs (SG Injection, SGI) and (b) from organic/inorganic PGs released from SG degradation in the troposphere (PG Injection, PGI). The annual tropical mean stratospheric chlorine input due to SGI is shown in Figure 3. Results are shown primarily from the BASE run (solid lines), though output from EXP2 (no Cl sink of VSLS) is also shown for C_2_Cl_4_ (dashed line, Figure 3c). Figure 3 also shows measurement‐based estimates of chlorine SGI (filled circles) derived from various high‐altitude aircraft observations (section 3.1). The open symbols show the model sampled at the measurement locations/times for the four most recent campaigns (although our model SGI estimates are reported as tropical annual means).

From the BASE run, we estimate that total chlorine SGI (Figure 3f) increased from ~55 (±10) ppt Cl in 2000 to ~93 (±17) ppt Cl in 2017. Of the present‐day total, CH_2_Cl_2_ is the largest contributor (~70%), followed by CHCl_3_ (~18%), C_2_H_4_Cl_2_ (~9%), and C_2_Cl_4_ (~2%). The observation data provide some constraint on the contribution from known potential “other” Cl‐VSLS that were not included in our model: for example, C_6_H_5_Cl and C_2_H_5_Cl. Although the data are limited, it suggests that the sum of chlorine in these three minor species is <1 ppt Cl around the tropopause and that they thus make a negligible contribution to chlorine SGI. Our total SGI estimate (Table 4) is at the upper limit of the 72 (50–95) ppt Cl SGI range reported in the 2014 Ozone Assessment (Carpenter et al., 2014) that was appropriate for the year 2012. This reflects that we consider the most recent VSLS trends, including increases in CH_2_Cl_2_ since that assessment. For comparison, our corresponding 2012 modeled SGI estimate is 76 (±13) ppt Cl, in reasonable agreement to Carpenter et al. (2014).

**Table 4:** Modeled Stratospheric Chlorine Input (ppt Cl) Due to Source Gas Injection, Product Gas Injection and Total

Species	Contribution to stratospheric chlorine (ppt Cl)			
	SGI	PGI (COCl_2_)	PGI (Cl_y_)	SGI + PGI
CH_2_Cl_2_	65.2 (±12.0)	2.0 (±0.2)	8.3 (±3.0)	75.5 (±15.2)
CHCl_3_	17.1 (±2.0)	2.5 (±0.4)	1.0 (±0.4)	20.6 (±2.8)
C_2_Cl_4_	1.9 (±0.6)	0.5 (±0.1)	1.2 (±0.4)	3.6 (±1.1)
C_2_H_4_Cl_2_	8.5 (±1.9)	‐	2.2 (±0.7)	10.7 (±2.6)
C_2_HCl_3_	<0.01	0.22 (±0.04)	0.26 (±0.05)	0.48 (0.09)
Total Cl	~93 (±17)	5 (±0.7)	13 (±4.6)	111 (±22)

*Note*. Average 2017 values (±1 standard deviation) are calculated from the BASE simulation. SGI = source gas injection; PGI = product gas injection.

From Figure 3, the model generally captures the measured total chlorine SGI well, which was observed to be similar during the 2013 ATTREX (102 ± 13 ppt Cl), 2014 ATTREX (105 ± 11 ppt Cl), and 2015 VIRGAS (104 ± 15 ppt Cl) missions. For example, when sampled at the measurement locations, the equivalent model estimate for the VIRGAS campaign is ~110 ppt Cl. The observed SGI from the most recent campaign, POSIDON in 2016, was ~120 (±27) ppt Cl, also in good agreement to our campaign‐sampled model (118 ± 6 ppt Cl). In principle, the relatively large chlorine SGI from the POSIDON mission, conducted in the West Pacific, could have been influenced by proximity to major VSLS source regions (e.g., Oram et al., 2017). However, we note that the campaign‐sampled model output—with no zonal variability in VSLS loading at the surface—agrees well with the measurement data. The implication of this is that spatiotemporal variability in (a) Cl‐VSLS troposphere‐to‐stratosphere transport and/or (b) Cl‐VSLS tropospheric lifetimes is a significant influence on regional SGI variability. This is also reflected in the difference between the model tropical average SGI (sold lines, Figure 3) versus the open circles (campaign‐sampled).

For individual SGs, the modeled SGI time series reflects known surface trends, including increases in CH_2_Cl_2_ since the mid‐2000s, relatively stable levels of CHCl_3_ until ~2010 following a small increase, and long‐term decreases in C_2_Cl_4_ (Figure S1). Despite our relatively simple approach to constraining these Cl‐VSLS at the surface, model‐measurement agreement is reasonable throughout the simulation period, particularly for CH_2_Cl_2_ and CHCl_3_, the most abundant compounds (see also Figures S3–S5). While aircraft measurements are somewhat sporadic and therefore should not be used in isolation as an indicator of robust long‐term trends, the observations in Figure 3 show the signature of increasing CH_2_Cl_2_ between 2004 and 2016. This corroborates the reported CH_2_Cl_2_ increases from surface records (e.g., Hossaini et al., 2017) and measurements in the upper troposphere (Leedham Elvidge et al., 2015). The “leveling off” of the model CH_2_Cl_2_ SGI time series in Figure 3a is driven by the model surface boundary conditions, based on surface observations, which in turn reveal an apparent slowdown in tropospheric CH_2_Cl_2_ growth. This is further discussed in section 4.5 in the context of total chlorine from VSLS. Note, in the case of C_2_H_4_Cl_2_, for which no time trend in the model was applied, the observed data do not appear to show a clear trend and suggest an SGI contribution of the order of ~5–15 ppt Cl in 2014–2016.

Switching off the Cl atom sink of individual VSLS made a negligible difference to the calculated chlorine SGI in our model, apart for C_2_Cl_4_. Modeled SGI from C_2_Cl_4_ is ~65% larger in EXP2 (without the C_2_Cl_4_ + Cl sink) relative to the BASE simulation, and this leads to significantly poorer agreement to the aircraft data. On this basis, the +Cl reaction should be included in models and the resulting lower lifetime (section 4.1) considered in relevant box‐model emission calculations.

### 4.3 PGI

**Figure 4:**
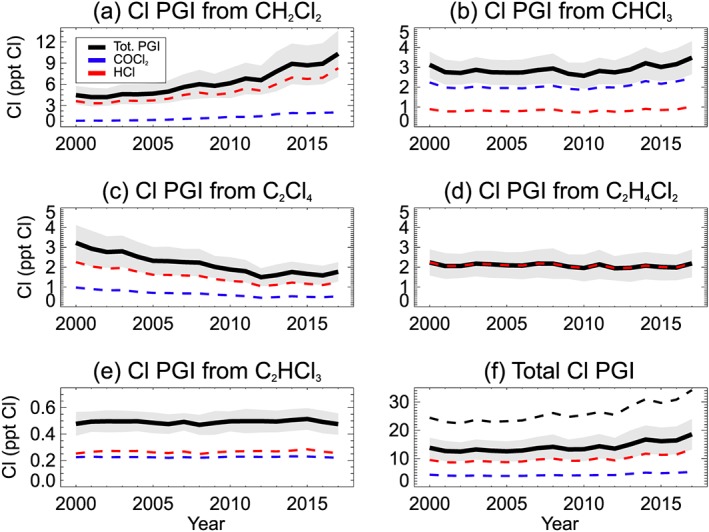
Modeled annual mean stratospheric chlorine product gas injection (ppt Cl) originating from (a) CH_2_Cl_2_, (b) CHCl_3_, (c) C_2_Cl_4_, (d) C_2_H_4_Cl_2_, (e) C_2_HCl_3_, and (f) total PGI. Dashed lines denote the PGI contribution from the idealized COCl_2_ (blue) and Cl_y_ (red) tracers. The solid lines denote the total PGI (i.e., the sum of COCl_2_ and Cl_y_ contributions), with shading denoting ±1 standard deviation from the mean. Model output is from experiment BASE, though in panel (f) the total PGI from EXP3 (upper limit for PGI) is also shown (dashed black line). PGI = product gas injection.

Figure 4 shows the modeled stratospheric chlorine PGI due to COCl_2_ and Cl_y_ from individual SGs (Figures 4a–4e), along with the total chlorine PGI from VSLS (Figure 4f). Degradation products from CH_2_Cl_2_ are the largest contributors to the overall PGI, accounting for ~57% of the total (Table 4). From the BASE simulation, we estimate chlorine PGI from CH_2_Cl_2_ increased from ~4.5 (±1.3) ppt Cl in 2000 to ~10.3 (±3.2) ppt Cl in 2017. Over the same period PGI due to C_2_Cl_4_ decreased by ~1.5 ppt Cl in response to its declining tropospheric abundance, offsetting some of this increase from CH_2_Cl_2_. In contrast, PGI due to CHCl_3_ products remained relatively stable over the simulation period (~3.5 ± 0.8 ppt Cl in 2017). Our best estimate of total chlorine PGI (BASE run) is ~13.8 (±3.5) ppt Cl in 2000, rising to ~18.6 (±5.2) ppt Cl in 2017. Of the present‐day PGI total, ~13 ppt Cl is in inorganic form (Cl_y_), in agreement with the 10 (0–20) ppt Cl estimate of Carpenter et al. (2014). However, our BASE run contribution from COCl_2_ (~5 ppt Cl) is a factor of 3 lower than their “best estimate” of 15 (0–30) ppt Cl, while within the large uncertainty range. In the supporting information, we discuss the uncertainties of our COCl_2_ simulation (Text S2) and compare modeled COCl_2_ profiles to ACE data (Figure S6).

Although our idealized model setup does not include a detailed, explicit treatment of tropospheric PG chemistry, a simple upper limit estimate for PGI is obtained from EXP3. This experiment assumed no tropospheric removal of COCl_2_ or Cl_y_ and is denoted in Figure 4f by the black dashed line. From EXP3, our upper limit of total PGI is ~34.2 (±7.4) ppt Cl in 2017, approximately 80% larger than the BASE run. Both the BASE and EXP3 model estimates are within the large uncertainty range (0–50 ppt Cl) of current assessments (Carpenter et al., 2014). Our analysis constrains the lower end of this range and shows that a nonzero PGI contribution is likely. However, we also note that chlorine PGI due to COCl_2_ (and hence total PGI) could be underestimated in our model. Both the BASE run and EXP3 underestimate measured COCl_2_ around the tropical tropopause from the ACE mission by around a factor of 3 (see supporting information). On the one hand, this does not necessarily imply an underestimation of COCl_2_ produced by Cl‐VSLS, given that COCl_2_ is also a product of long‐lived CCl_4_ and CH_3_CCl_3_ degradation (section 2.3). On the other, we note that EXP8, which had a larger assumed fixed yield of COCl_2_ production from CH_2_Cl_2_ (see section 2.4), provides much better agreement to the ACE measurements (Figure S6). In summary, factoring in the full range of simulations performed here, modeled chlorine PGI due to VSLS‐produced COCl_2_ is ~5 ppt Cl (BASE run, Table 4) and ~18 ppt Cl (EXP8) in 2017; that is, uncertain by a factor of >3.

### 4.4 Total Stratospheric Chlorine Injection and Trends

**Figure 5:**
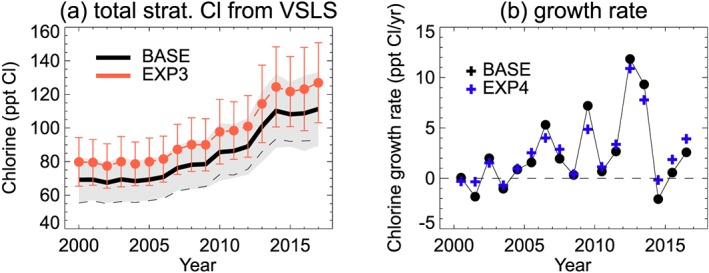
Time series of modeled (a) total stratospheric chlorine input from Cl‐VSLS (ppt Cl) and (b) the corresponding growth rate (ppt Cl/year). In panel (a) model output is from the BASE simulation and shows the annual average (black line) total stratospheric chlorine injection (i.e., the sum of source and product gas contributions) with ±1 standard deviation (gray shading). Note, the thin dashed line (black) shows the BASE contribution from source gases only. Also shown is the total stratospheric chlorine injection from EXP3 (orange line, product gas injection upper limit). In panel (b) output from EXP4 (repeating meteorology) is shown in addition to the BASE simulations. VSLS = very short‐lived substances.

Figure 5 shows the evolution of total stratospheric chlorine (VSLCl_tot_) from VSLS (i.e., the sum of SGI and PGI contributions) over the full simulation period. From the BASE run, we estimate that VSLCl_tot_ increased from ~69 (±14) ppt Cl in 2000, to ~111 (±22) ppt Cl in 2017: that is, a ~61% increase over the 18‐year period. Chlorine from CH_2_Cl_2_ is the largest contributor to VSLCl_tot_ accounting for ~68% of the total, followed by CHCl_3_ (~19%), C_2_H_4_Cl_2_ (~10%), and C_2_Cl_4_ (~3%). The contribution from C_2_HCl_3_ is negligible. For individual species, the relative importance of SGI versus PGI varies (Table 4), though SGI dominates VSLCl_tot_ overall, accounting for >80% of the total. This is due to modest relative PGI contributions (<20%) from the principal compounds CH_2_Cl_2_ and CHCl_3_, which have the longest tropospheric lifetimes (e.g., Table 1). Our BASE run estimate of VSLCl_tot_ (111 ± 22 ppt Cl in 2017) is slightly larger than the best estimate of 95 (50–145) ppt Cl reported by Carpenter et al. (2014), though within their range of uncertainty. The difference between these estimates reflects the different periods under consideration. Our estimate is an up‐to‐date assessment based on the most recent observed Cl‐VSLS surface trends.

Results from model run EXP3 provide an upper limit to VSLCl_tot_ from our study (see Figure 5a). This experiment does not include tropospheric wet removal of COCl_2_ or Cl_y_ PGs and thus assumes their efficient transport to the stratosphere, once produced. From EXP3, VSLCl_tot_ is 126 (±24) ppt Cl in 2017, that is, ~14% larger than the BASE model. The results from this and all other sensitivity experiments are summarized in Figure S7. We find that VSLCl_tot_ is fairly insensitive to changes in tropospheric [OH]; increasing/decreasing [OH] by 25% (EXP5 and EXP6) leads to only a ~2% decrease/increase in VSLCl_tot_. Comparing EXP4 (repeating 2000 meteorology) to the BASE run (evolving meteorology) shows a relatively small influence of interannual meteorological variability, with VSLCl_tot_ ~1% larger in the former run. While this analysis is not exhaustive, details of tropospheric transport processes will be less important for Cl‐VSLS (e.g., CH_2_Cl_2_ and CHCl_3_) compared to analogous brominated compounds (e.g., Liang et al., 2014; Tegtmeier et al., 2012), owing to much longer tropospheric lifetimes of the former. Similarly, the importance of Cl atoms as a C_2_Cl_4_ sink notwithstanding (Figure S7c and section 4.2), the overall influence of Cl atom oxidation of Cl‐VSLS on VSLCl_tot_ is small (<0.5%).

Of all parameters examined in the sensitivity experiments, the largest influence on VSLCl_tot_ comes from changing the CH_2_Cl_2_ surface boundary condition (SG_MF_) from one derived from NOAA data (BASE run) to one derived from AGAGE (EXP1). In the tropical band 0–30°N, the latter is ~19 ppt lower in 2016 (Figure S1) and its use translates to a ~18% decrease in VSLCl_tot_ overall (EXP1 relative to BASE). These differences between the two networks for CH_2_Cl_2_ have been previously noted and likely reflect differences in calibration scales (of the order of ~10%) and the different sampling locations (Carpenter et al., 2014). A detailed examination of differences between the measurement networks is beyond the scope of the paper. However, in terms of sampling location within the 0–30°N band, we note that NOAA data are obtained from a Pacific site, while AGAGE data are from an Atlantic one. As noted in section 3, aircraft measurements of CH_2_Cl_2_ at the point of stratospheric entry (reported on a scale consistent with NOAA) are well reproduced by our BASE model.

Modeled growth rates of VSLCl_tot_, while found to be highly variable (Figure 5b), remained positive year‐to‐year in the period 2004/2005 to 2013/2014. The start of this period corresponds roughly to the onset of the recently observed increases in CH_2_Cl_2_ (e.g., Hossaini et al., 2015a). We find that post 2014, VSLCl_tot_ growth rates turned negative for the first time since prior to 2004; for the 2014/2015 period the VSLCl_tot_ growth rate was −2.1 ppt/year Cl in the BASE simulation. In subsequent years, growth was positive at 0.6 ppt Cl/year for 2015/2016 and 2.6 ppt Cl/year for 2016/2017; small in comparison to growth in the 2012/2013 and 2013/2014 periods, for example. The relative slow growth in these most recent years mainly reflects a stabilization in CH_2_Cl_2_, which is present in the surface boundary conditions forcing the model (Figure S1). This can be seen in the leveling off of the modeled CH_2_Cl_2_ abundance in the tropical upper troposphere (Figure 3). Continued CH_2_Cl_2_ observations in coming years will be required to assess whether the above is a transient effect, or rather a longer‐term stabilization of CH_2_Cl_2_ mole fractions/emissions. However, we note that in the context of the full simulation period (Figure 5b), sporadic years of near‐zero VSLCl_tot_ growth are somewhat typical, and that the apparent slower growth in the most recent years is also slightly influenced by meteorology (i.e., comparing the blue and black data in Figure 5b).

Although the contribution of VSLS to stratospheric chlorine has increased in absolute terms since the early 2000s, it remains small in comparison to the chlorine provided by long‐lived halocarbons (i.e., CFCs, HCFCs, CCl_4_, CH_3_Cl, etc.). A simple estimate of stratospheric chlorine from long‐lived halocarbons is presented in Figure S8. This is based on the surface abundance of the major compounds provided by the WMO A1 scenario (WMO, 2014), and the assumption that 100% of the surface chlorine from these compounds reaches the stratosphere. From the BASE simulation, we estimate that Cl‐VSLS accounted for ~2% of stratospheric chlorine in 2000, rising to ~3.4% in 2017. This figure will likely increase further if Cl‐VSLS abundances increase in coming years, against a backdrop of declining chlorine from long‐lived compounds following the A1 scenario.

### 4.5 Influence of Chlorinated VSLS on Stratospheric HCl Trends

In the previous section, we discussed the temporal evolution of stratospheric chlorine from VSLS and time‐varying growth rates. An ordinary least squares fit to the full time series of data in Figure 5a (2000–2017) suggests total chlorine from VSLS (VSLCl_tot_) increased at a mean rate of ~2.9 (±0.3) ppt Cl/year. Over the shorter 2004–2017 period, roughly corresponding to the onset of significant CH_2_Cl_2_ growth in the mid‐2000s, the trend is 3.8 (±0.3) ppt Cl/year—in good agreement with our earlier estimates (Hossaini et al., 2015b). Note that changes to CH_2_Cl_2_ dominate the trend in VSLCl_tot_. For example, without CH_2_Cl_2_ included, the 2004–2017 trend is reduced to ~0.23 (±0.06) ppt Cl/year, reflecting small increases in CHCl_3_ over the period (e.g., Figure 3b).

To provide additional “top down” constraints on the magnitude of historical trends in stratospheric chlorine from VSLS, we consider satellite observations (section 3.2) of stratospheric HCl (2004–2017) from the ACE satellite mission (Bernath & Fernando, 2018). Our analysis focusses on the upper stratosphere (pressures less than ~10 hPa) where HCl is far less affected by dynamical variability relative to the lower stratosphere (e.g., Stolarski et al., 2018). Four additional integrations of the stratospheric configuration of TOMCAT/SLIMCAT were performed. The first stratospheric simulation (referred to as “S‐NOVSLS”) contained no chlorine from VSLS. The second (S‐BASE) considered time‐dependent stratospheric loadings of CHCl_3_, CH_2_Cl_2_, C_2_Cl_4_, and C_2_H_4_Cl_2_ (Figure 3) and their associated PGs (Figure 4), based on values from the BASE simulation. As the dynamics are the same in both runs, the impact of VSLS growth on HCl trends can be isolated readily. The third stratospheric simulation (S‐EXP3) was identical to the above but stratospheric VSLS loadings were taken from EXP3 (i.e., PGI upper limit). Finally, S‐FIXDYN was identical to S‐BASE but employed repeating year‐2000 dynamics. This allowed us to examine the relative influence of chemistry versus dynamics on simulated HCl trends. All simulations were performed for the period 2000 to 2017. The version of the stratospheric model employed here was recently used by Chipperfield et al. (2018) to investigate lower stratospheric ozone trends and is used by Harrison et al. (2018) to interpret long‐term COCl_2_ observations from ACE. It has also been extensively evaluated in terms of both chemistry and transport (e.g., Harrison et al., 2016; Wales et al., 2018).

**Figure 6:**
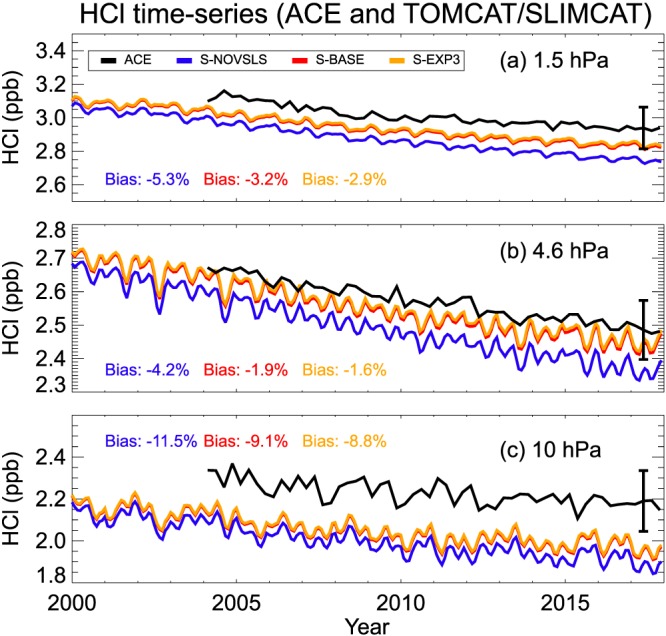
Modeled versus observed HCl (ppb) at (a) 1.5, (b) 4.6, and (c) 10 hPa between 60°N and 60°S latitude. The observed satellite data are from the ACE instrument (section 3.2). Annotated biases (model − observation, %) are shown for experiments S‐BASE (red), S‐EXP3 (orange), and S‐NOVSLS (blue). The vertical error bar (shown illustratively for 2017) represents the 1σ ACE HCl measurement error, ~0.12 ppb.

**Figure 7:**
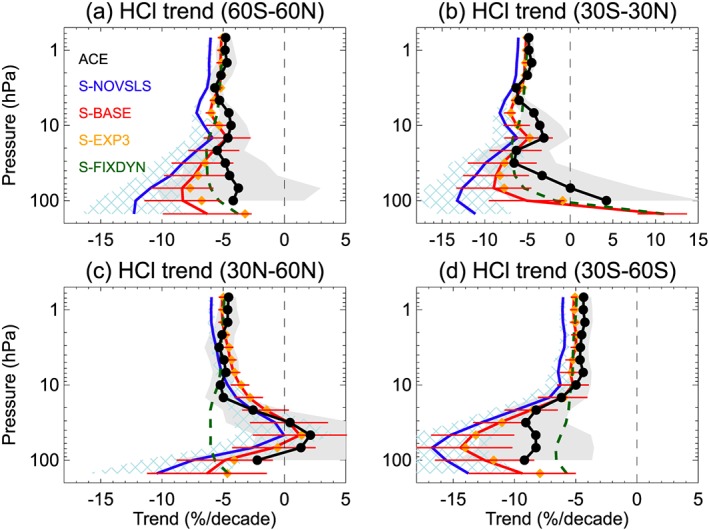
Comparison of stratospheric HCl trends (% per decade) from ACE satellite observations (Bernath & Fernando, 2018) and model runs (2004–2017). Mean trends are for (a) 60°S to 60°N, (b) 30°S to 30°N, (c) 30 to 60°N, and (d) 30 to 60°S. Horizontal error bars on model run S‐BASE denote 2σ errors, also represented by hatching and gray shading.

Figure 6 compares the modeled upper stratospheric HCl time series (60°S–60°N) to that observed by ACE. The model‐measurement agreement is generally very good, with biases (see annotations on figure) ~12% or less. Model runs with additional chlorine from VSLS included (runs S‐BASE and S‐EXP3) exhibit a lower model‐measurement bias (relative to run S‐NOVSLS) by several percent. However, we also note the size of the ACE measurement error is comparable to VSLCl_tot_ in 2017 (around ±0.12 ppb). Bernath and Fernando (2018) provided an up‐to‐date assessment of stratospheric HCl trends based on these ACE data. Between 60°S and 60°N, they estimated a mean rate of HCl decline of −4.8 (±0.7)% per decade in the upper stratosphere (pressures less than 1.5 hPa, ~45–51 km) between 2004 and 2017. In the upper stratosphere, HCl is most abundant, provides a proxy for total atmospheric chlorine, and is where the trend is least uncertain. The vertically resolved ACE HCl trends for different latitudes and equivalent model estimates are given in Figure 7. Note, in order to perform a like‐for‐like comparison to the model, the ACE trend data here does not incorporate N_2_O into the trend analysis (Bernath & Fernando, 2018).

Figure 7 highlights the major influence of dynamical variations (i.e., comparing S‐FIXDYN and S‐BASE) in the lower stratosphere and a contrasting dynamical influence in the two midlatitude regions (Figure 7c vs 7d). In the midlatitude lower stratosphere, the sign and magnitude of the model trends (e.g., S‐BASE) agree reasonably well to those from ACE considering the large uncertainty. Away from the lower stratosphere (at higher altitude), HCl trends are far less sensitive to dynamical variability (e.g., Stolarski et al., 2018), which is apparent from the model data in Figure 7 (i.e., the convergence of the red and dashed green lines above 10 hPa).

In the upper stratosphere (pressure ≤10 hPa), the modeled and observed trends are in reasonable agreement. Note, at pressures less than ~5 hPa, the 2σ error bars do not overlap for the with/without VSLS trend estimates from the model. Between 60°S and 60°N, the mean HCl trend in the upper stratosphere is −5.0 (±0.3)% per decade from S‐FIXDYN, in close agreement (~4% larger) to the ACE trend of −4.8 (±0.7)% per decade (Table S3). HCl trends from S‐BASE (−5.2 ± 0.3% per decade) and S‐NOVSLS (−6.1 ± 0.2% per decade) are ~8% and ~27% larger the ACE value, respectively. The difference between these two model runs reveals a 15% slower rate of upper stratospheric HCl decline, attributable solely to the inclusion of Cl‐VSLS. The S‐NOVSLS trend is in close agreement to the observed rate of change in total tropospheric chlorine measured by NOAA (−6.1 ± 0.09% per decade for 2004–2016) when excluding Cl‐VSLS, and with a 7‐year time lag applied to the NOAA time series (Figure S9) to account for transport time to the upper stratosphere. The HCl trend from S‐EXP3, with a greater loading of chlorine from Cl‐VSLS relative to S‐BASE, is also shown in Figure 7. In the upper stratosphere, the S‐EXP3 trend (−5.1 ± 0.3% per decade) is similar to the BASE run.

Overall, the above comparisons show that the model provides a reasonable representation of observed stratospheric HCl trends, including when Cl‐VSLS are considered. While we acknowledge that HCl trends from models and measurements are uncertain, the upper stratospheric trend analysis provides further evidence that VSLS may have offset some of the possible benefits of the Montreal Protocol in reducing stratospheric chlorine over the last decade. Our simulations indicate that upper stratospheric HCl has declined at a 15% slower rate globally over the 2004–2017 period due to VSLS, relative to what would be expected in their absence.

## 5 Summary and Concluding Remarks

We have performed 3‐D CTM simulations and analyzed observation data to (a) quantify the stratospheric input of chlorine from VSLS (CH_2_Cl_2_, CHCl_3_, C_2_Cl_4_, C_2_H_4_Cl_2_, and C_2_HCl_3_) between 2000 and 2017, (b) quantify its sensitivity to a range of factors (including assumptions in model chemistry and model transport), and (c) examine if inclusion of Cl‐VSLS improves the model representation of stratospheric HCl trends. Our main findings and recommendations for future research are summarized below:

1. Stratospheric chlorine SGI from VSLS increased from ~55 (±10) ppt Cl in 2000 to ~93 (±17) ppt Cl in 2017. CH_2_Cl_2_ accounts for ~70% of the present‐day total. Observations from NASA campaigns (2004–2016) show increasing CH_2_Cl_2_ around the tropical tropopause, consistent in magnitude to modeled increases and corroborating reported increases from independent surface (Hossaini et al., 2015a; Hossaini et al., 2017) and upper tropospheric (Leedham Elvidge et al., 2015) records. Total chlorine SGI observed during the 2015 VIRGAS (104 ± 15 ppt Cl) and 2016 POSIDON missions (120 ± 27 ppt Cl) agree well with our model (to within a few percent, when the model is sampled during the campaign periods). Total SGI is insensitive to inclusion of tropospheric Cl atom oxidation of VSLS in our model (when [Cl] = 1.3 × 10^3^ atoms per cubic centimeter). However, the C_2_Cl_4_ + Cl reaction is significant, with its inclusion (a) reducing the C_2_Cl_4_ lifetime by up to ~50% at 10 km and (b) greatly improving C_2_Cl_4_ model‐measurement agreement around the tropopause.
2. As is the case with brominated VSLS, estimation of stratospheric PGI is challenging owing to the complexity of the chemical processes involved. Our simulations considered total PGI from COCl_2_ (produced by CHCl_3_, CH_2_Cl_2_, and C_2_Cl_4_) and from an idealized Cl_y_ tracer (produced by all), both of which had prescribed tropospheric lifetimes based on the literature. We estimate a nonzero chlorine PGI from VSLS of ~18 (±5) ppt Cl in 2017, within the 0–50 ppt Cl range reported in WMO (2014). When assuming no tropospheric wet removal of COCl_2_ and Cl_y_, this estimate increases to ~34 (±7) ppt Cl. Our parameterized scheme suggests COCl_2_ is a product of CH_2_Cl_2_ degradation, particularly in low NO_x_ regions. However, modeled COCl_2_ near the tropical tropopause is underestimated by a factor of ~3 and further work to elucidate the uncertain mechanism of COCl_2_ production is needed.
3. Total stratospheric Cl from VSLS (VSLCl_tot_) increased by ~61% between 2000 (~69 ± 14 ppt Cl) and 2017 (~111 ± 22 ppt Cl). In the same years, VSLS represented ~2% and ~3.4% of overall stratospheric chlorine—a small but significant portion that should increase given the ongoing decline of long‐lived chlorine (e.g., CFCs) under the Montreal Protocol. A sensitivity experiment in which PGs from VSLS were not removed in the troposphere by deposition processes gives a 2017 VSLCl_tot_ upper limit of 126 (±24) ppt Cl. We estimate VSLCl_tot_ increased by 3.8 (±0.3) ppt Cl/year between 2004 and 2017, with CH_2_Cl_2_ changes dominating the trend. Growth was negative or slow in the period 2014/2015 to 2016/2017, relative to much larger growth in 2012/2013 and 2013/2014. Further observations to determine whether this is a transient effect are needed.
4. Increasing stratospheric chlorine from VSLS between 2004 and 2017 will have likely had a small but significant influence on HCl trends in the upper stratosphere. Measurements from the ACE satellite mission show a mean rate of upper stratospheric HCl decline of −4.8% per decade between 2004 and 2017, compared to model trends of −5.1 to −5.2% per decade with VSLS, and −6.1% per decade without.

In conclusion, the contribution of VSLS to total stratospheric chlorine remains small in both absolute and relative terms. However, our results provide evidence that VSLS have acted to offset the rate of chlorine decline in the stratosphere, and therefore some of the possible benefits of the Montreal Protocol since the mid‐2000s. Given suggestions that anthropogenic CH_2_Cl_2_ emissions from major economies will increase in the coming decade (Feng et al., 2018), such an offset may continue.

## Supporting information

Supporting Information S1
