# Supplementary material for: Recent Trends in Stratospheric Chlorine From Very Short‐Lived Substances
Source: J Geophys Res Atmos. 2019 Feb 16;124(4):2318–35. doi: 10.1029/2018JD029400 (PMC6446807; doi:10.1029/2018JD029400)
Supplement: Supplementary file 1 — Supporting Information S1 [file JGRD-124-2318-s001.pdf]

AGU PUBLICATIONS

Journal of Geophysical Research

Supporting Information for

Recent Trends in Stratospheric Chlorine from Very Short-Lived Substances

Ryan Hossaini[1], Elliot Atlas[2], Sandip S. Dhomse[3], Martyn P. Chipperfield[3], Peter F. Bernath[4,5], Anton M. Fernando[6], Jens Mühle[7], Amber A. Leeson[1], Stephen A. Montzka[8], Wuhu Feng[3,9], Jeremy J. Harrison[10,11], Paul Krummel[12], Martin K. Vollmer[13], Stefan Reimann[13], Simon O'Doherty[14], Dickon Young[14], Michela Maione[15], Jgor Arduini[15] and Chris R. Lunder[16]

[1]Lancaster Environment Centre, Lancaster University, Lancaster, UK.

[2]University of Miami, Florida, USA.

[3]School of Earth and Environment, University of Leeds, Leeds, UK.

[4]Department of Chemistry and Biochemistry, Old Dominion University, Norfolk, Virginia, USA.

[5]Department of Chemistry, University of Waterloo, Waterloo, ON, Canada

[6]Department of Physics, Old Dominion University, Norfolk, Virginia, USA.

[7]Scripps Institution of Oceanography, University of California San Diego, La Jolla, California, USA.

[8]National Oceanic and Atmospheric Administration (NOAA), Boulder, Colorado, USA.

[9]NCAS, University of Leeds, Leeds, UK.

[10]Department of Physics and Astronomy, University of Leicester, Leicester, UK.

[11]National Centre for Earth Observation, University of Leicester, Leicester, UK.

[12]Climate Science Centre, CSIRO Oceans and Atmosphere, Aspendale, Victoria, Australia.

[13]Laboratory for Air Pollution & Environmental Technology, Empa, Swiss Federal Laboratories for Materials Science and Technology, Duebendorf, Switzerland.

[14]School of Chemistry, University of Bristol, Bristol, UK.

[15]Department of Pure and Applied Sciences, University of Urbino, Urbino, Italy

[16]Norwegian Institute for Air Research, Kjeller, Norway.

Corresponding author: Ryan Hossaini (r.hossaini@lancaster.ac.uk)

Contents of this file

Text S1 and S2
Figures S1-S9
Tables S1-S3

Introduction

This Supporting Information to the main article consists of two sets of supplementary text (S1-2), nine supplementary figures (S1-S11) and three supplementary tables (S1-S3).

Text S1.

To constrain the surface abundance of Cl-VSLS in our model, each compound (CH_2Cl_2, $CHCl_3$, C_2Cl_4, $C_2H_4Cl_2$ and C_2HCl_3) is prescribed a latitude-dependent mixing ratio boundary condition (Figure S1). Five latitude bands are considered for all species. Apart from $C_2H_4Cl_2$ and C_2HCl_3, the surface boundary condition varied annually. In our BASE simulation, the CH_2Cl_2 and C_2Cl_4 surface boundary conditions are based on data from the NOAA global monitoring network [e.g. Montzka et al., 2018]. Indicated below are the NOAA sites from which data were obtained, for each of the latitude bands. A summary of the NOAA sites and site codes can be found at: https://www.esrl.noaa.gov/gmd/dv/site/

- 60-90°N: ALT, SUM and BRW
- 30-60°N: MHD, LEF, HFM, THD and NWR
- 00-30°N: KUM and MLO
- 00-30°S: SMO
- 30-90°S: CGO, PSA and SPO

In our BASE simulation, the $CHCl_3$ surface boundary condition is based on data from the AGAGE network [e.g. Prinn et al., 2000]. Indicated below are the AGAGE sites that were used.

- 60-90°N: ZEP
- 30-60°N: MHD, THD, JFJ and CMN
- 00-30°N: RPB
- 00-30°S: SMO
- 30-90°S: CGO

For $C_2H_4Cl_2$ and C_2HCl_3, no long-term surface monitoring data exists. Therefore, their surface boundary condition was estimated based on HIPPO data [Wofsy et al., 2011] and did not vary annually. For $C_2H_4Cl_2$: 15 ppt (60-90°N), 15 ppt (30-60°N), 10 ppt (00-30°N), 3 (00-30°S) and 2 ppt (30-90°S). For C_2HCl_3: 0.5 ppt (60-90°N), 0.5 ppt (30-60°N), 0.5 ppt (00-30°N), 0.2 (00-30°S) and 0.2 ppt (30-90°S).

Text S2.

To assess the fidelity of $COCl_2$ production in the model, Figure S6 compares tropical mean profiles of total $COCl_2$ to measurements from the ACE satellite mission (available in 2014 and 2015, Section 3.2). Total $COCl_2$ includes production in the model from (a) Cl-VSLS, (b) carbon tetrachloride (CCl_4) and (c) methyl chloroform. The latter two compounds are well established $COCl_2$ sources [e.g., Kindler et al., 1995] and dominate its production above 20 km. This is evident from the dashed black profile in Figure S6 which shows tagged $COCl_2$ from these long-lived precursors only. Considering all $COCl_2$ sources, the BASE model captures the general features of the observed $COCl_2$ profiles, including the rapid $COCl_2$ increase in the lower stratosphere and a maximum at ~24 km (Figure S6). However, it is also apparent that $COCl_2$ is underestimated by the model at around 18 km and below (in the upper part of the tropical tropopause layer), where modelled $COCl_2$ falls outside of the measurement error bars. Such an underestimation has been reported in our previous modelling work [Hossaini et al., 2015b] that examined ACE $COCl_2$ in earlier years, and remains unexplained. Even when no tropospheric $COCl_2$ removal is assumed (EXP3), $COCl_2$ is underestimated in this region by a factor of ~3 (a marginal improvement over the BASE model). However, a substantial reduction in this bias is achieved by EXP8, from which modelled/measured $COCl_2$ agree to within a factor of 2 or less between 15-18 km; within the ranges of uncertainty. Recall, EXP8 assumes a fixed yield of $COCl_2$ from CH_2Cl_2 degradation of unity, as opposed to calculating the yield interactively based on Equation 1. We are not suggesting that EXP8 is more realistic in its treatment of $COCl_2$ production from CH_2Cl_2, but this comparison indicates that the mechanism and yield of $COCl_2$ production requires further examination. In any case, improved agreement from EXP8 in the lower stratosphere appears to come at the expense of the agreement at higher altitudes in 2015 (Figure S6). It should also be noted that none of the model simulations fully captures the observed vertical gradient in $COCl_2$ below the ~24 km peak.

In an assessment of product gases from Cl-VSLS, Ko and Poulet et al. [2003] did not consider $COCl_2$ as a major product of CH_2Cl_2 degradation. In our BASE model, $COCl_2$ from CH_2Cl_2 provides a stratospheric chlorine PGI (~2 ppt Cl) similar to that from $CHCl_3$ (Table 4). Recall that production of $COCl_2$ from CH_2Cl_2 is calculated in a semi-explicit fashion using Equation 1. Figure S6c shows the modelled $COCl_2$ yield expressed as an annual mean at the surface. We find the lowest values generally occur over industrialized regions (typically <0.2), where elevated NO_x levels act to suppress $COCl_2$ production. This occurs because NO_x competes with HO_2 in the model for reaction with the $CHCl_2O_2$ peroxy radical (see discussion in Section 2.3). Over large areas of the ocean the yield is ~0.5, with a mean of ~0.3 over the entire tropics (±20° latitude); i.e. a substantially lower value than the fixed yield of 1 assumed in EXP7 and EXP8. These results suggest that CH_2Cl_2 may be a significant $COCl_2$ source, in addition to $COCl_2$ produced from $CHCl_3$ and C_2Cl_4, particularly in low NO_x regions.

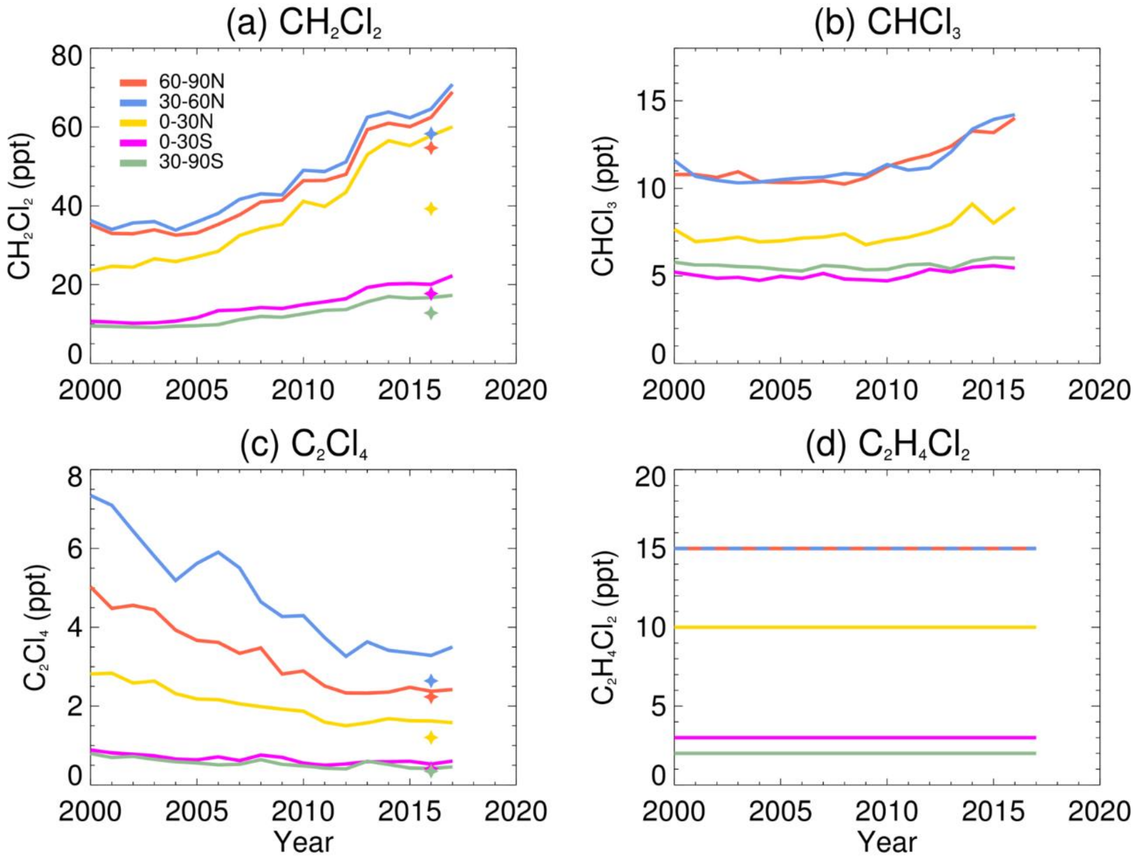

Figure S1. Latitude-dependent surface mixing ratio boundary condition (ppt) for CH_2Cl_2, $CHCl_3$, C_2Cl_4 and $C_2H_4Cl_2$ (ppt). For CH_2Cl_2 and C_2Cl_4, estimates are based on NOAA data (lines) and, in 2016 only, AGAGE (filled stars). $CHCl_3$ data based on AGAGE network (lines). $C_2H_4Cl_2$ data is non time-dependent and is estimated (see Text S1).

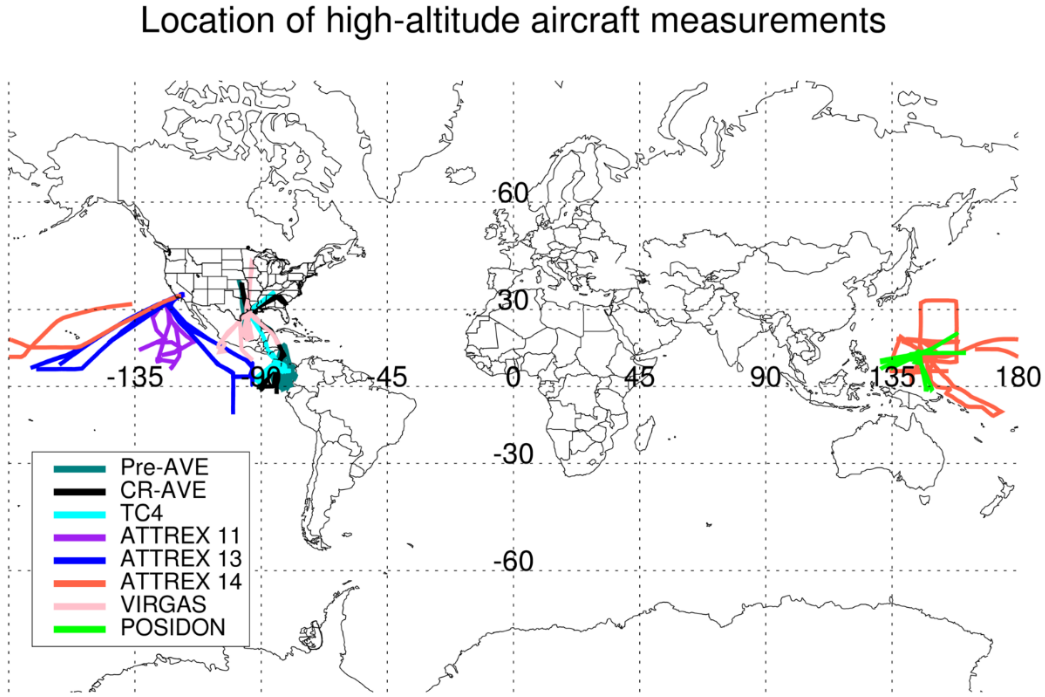

Figure S2. Location of the aircraft campaigns considered in this study.

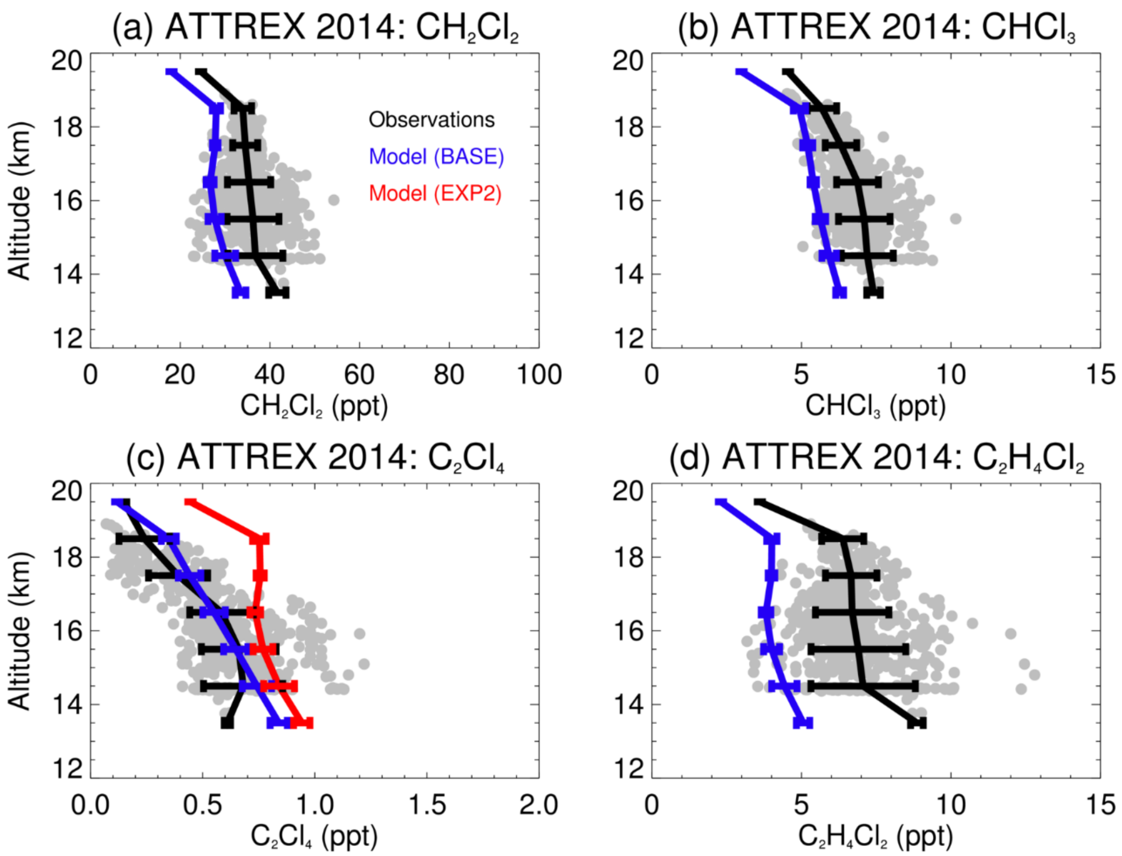

Figure S3. Observed and modelled profiles of (a) CH_2Cl_2, (b) $CHCl_3$, (c) C_2Cl_4 and (d) $C_2H_4Cl_2$ mixing ratio (ppt). All observations (grey circles) from the 2014 ATTREX field campaign (see Figure S2 also) are shown. The black (observations) and blue (model, BASE run) lines are the mean profiles (±1σ) over the campaign in 1 km altitude bins. Model data from EXP2 for C_2Cl_4 (i.e. no C_2Cl_4 + Cl sink).

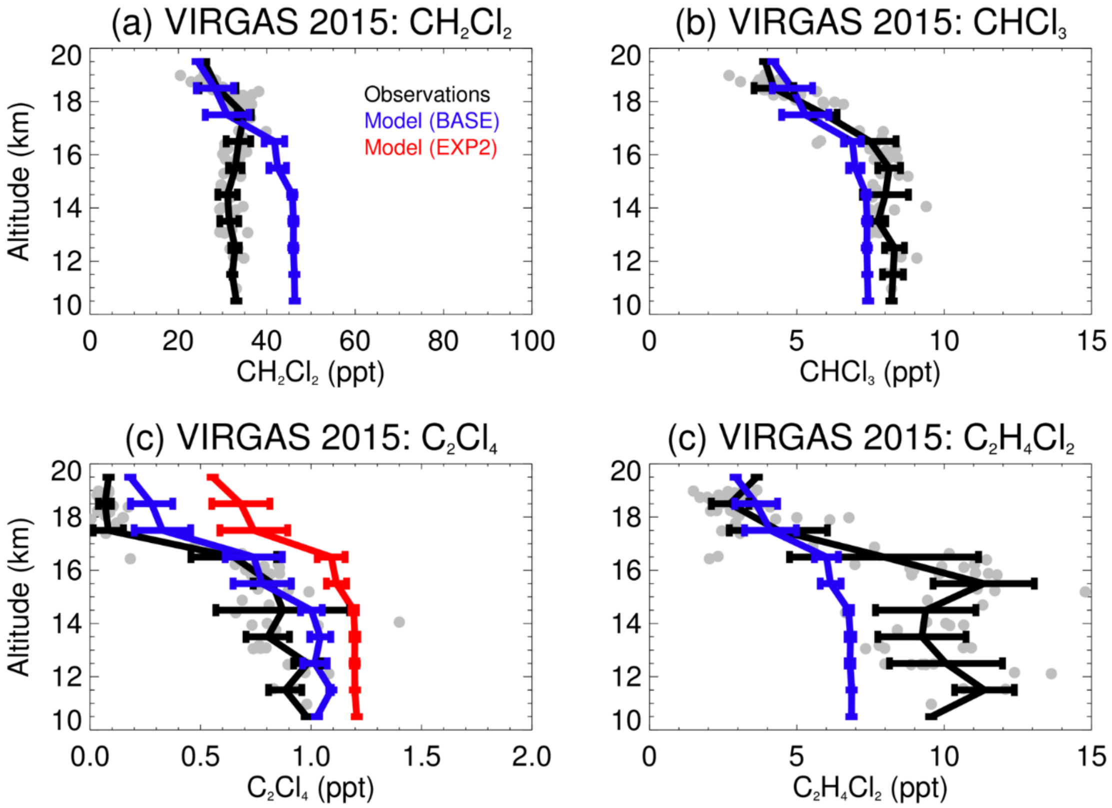

Figure S4. As Figure S3 but for the 2015 VIRGAS mission.

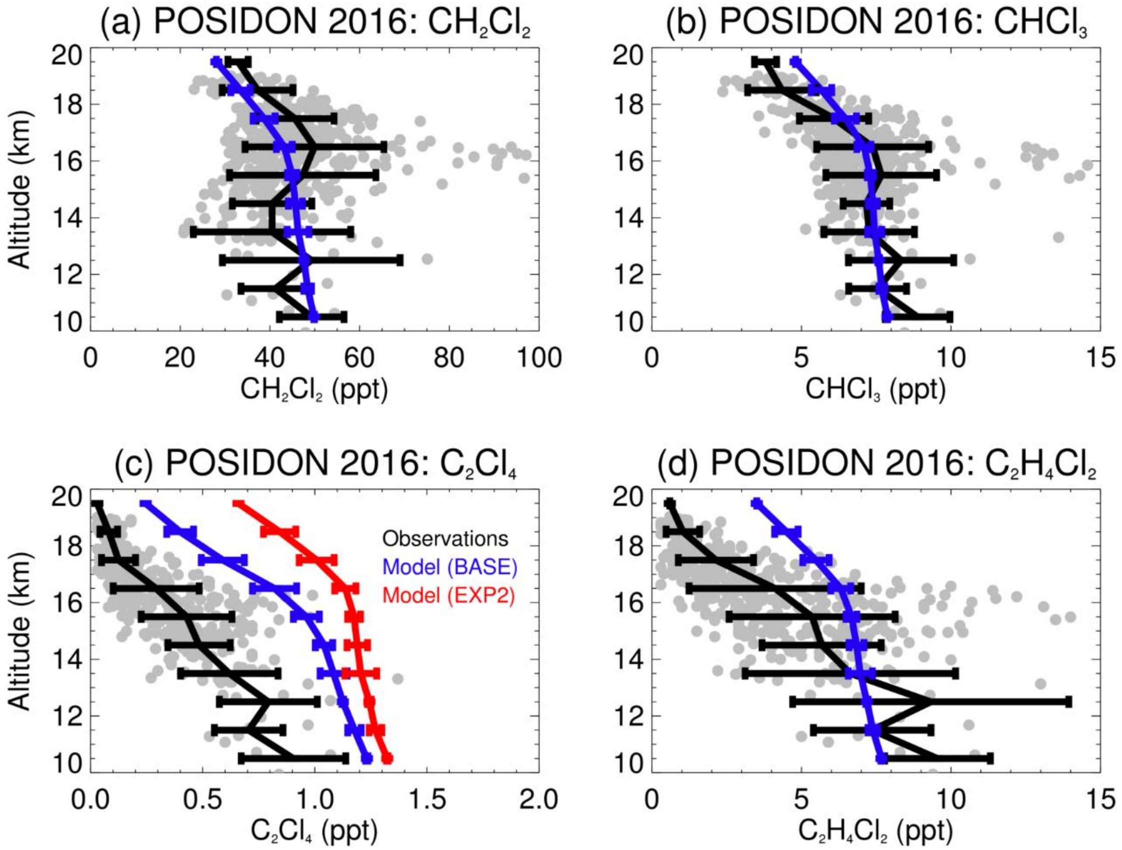

Figure S5. As Figure S3 but for the 2016 POSIDON mission.

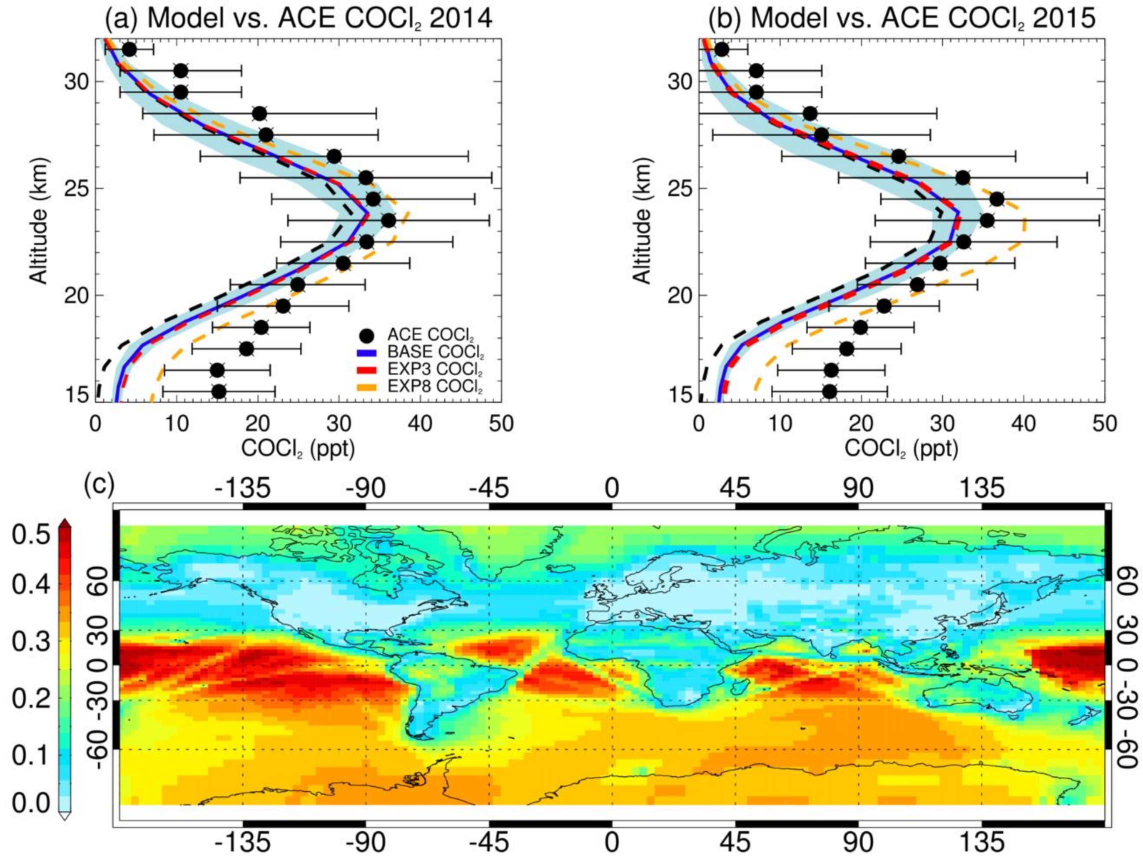

Figure S6. Comparison of modelled and observed tropical mean $COCl_2$ profiles (ppt) for (a) 2014 and (b) 2015. Median $COCl_2$ observations are from the ACE satellite mission (Section 3.2). Horizontal bars denote the median absolute deviation. Output from model simulations BASE (shading denotes ±1σ from the mean) and EXP3 shown. Dashed black lines show modelled $COCl_2$ from long-lived CCl_4 and CH_3CCl_3 only. Panel (c) shows the modelled $COCl_2$ yield (Y) from CH_2Cl_2 degradation at the surface (Section 2.3.1) computed from Equation 1 (as used in BASE run).

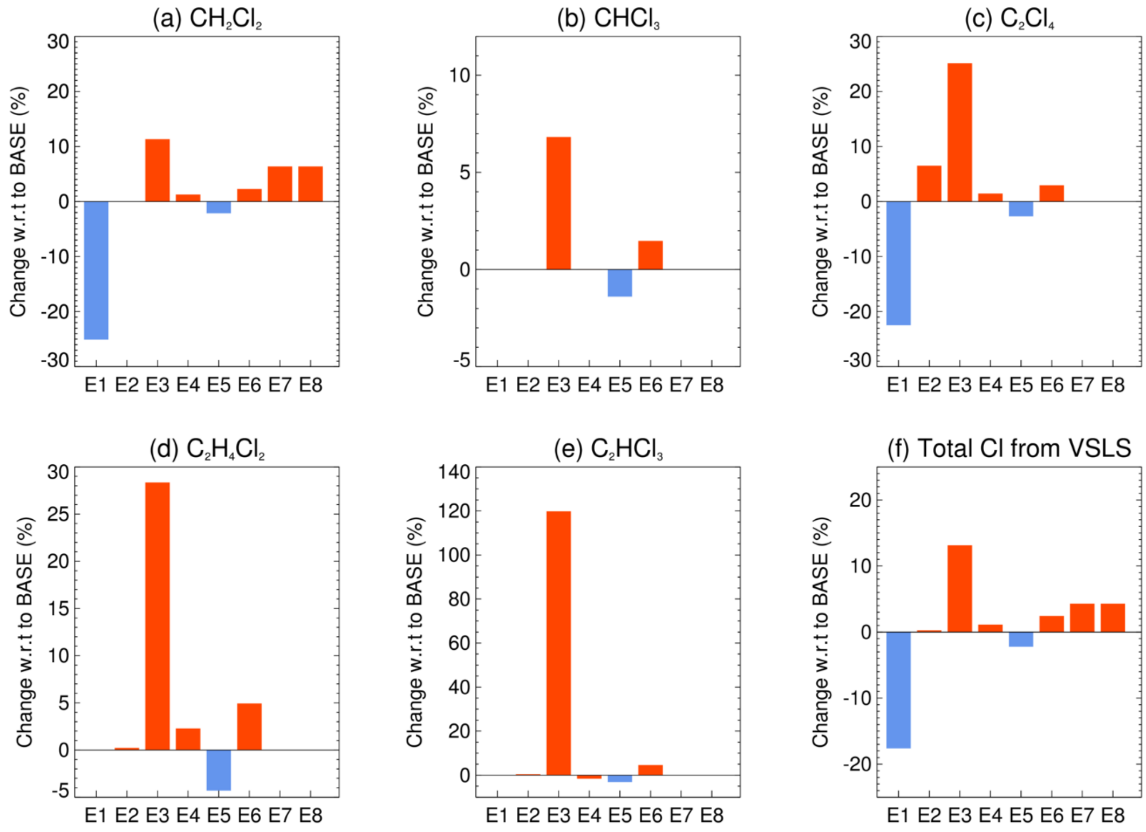

Figure S7. Results of sensitivity experiments showing the change (%) in stratospheric chlorine from VSLS with respect to the BASE run. The change in stratospheric chlorine from individual VSLS are shown (panels a-e) as is the change in total stratospheric chlorine from VSLS (panel e). The different sensitivity experiments (Table 1, main article) are labelled on the x-axis. Percent changes calculated as E# – BASE. Note the different scales between panels.

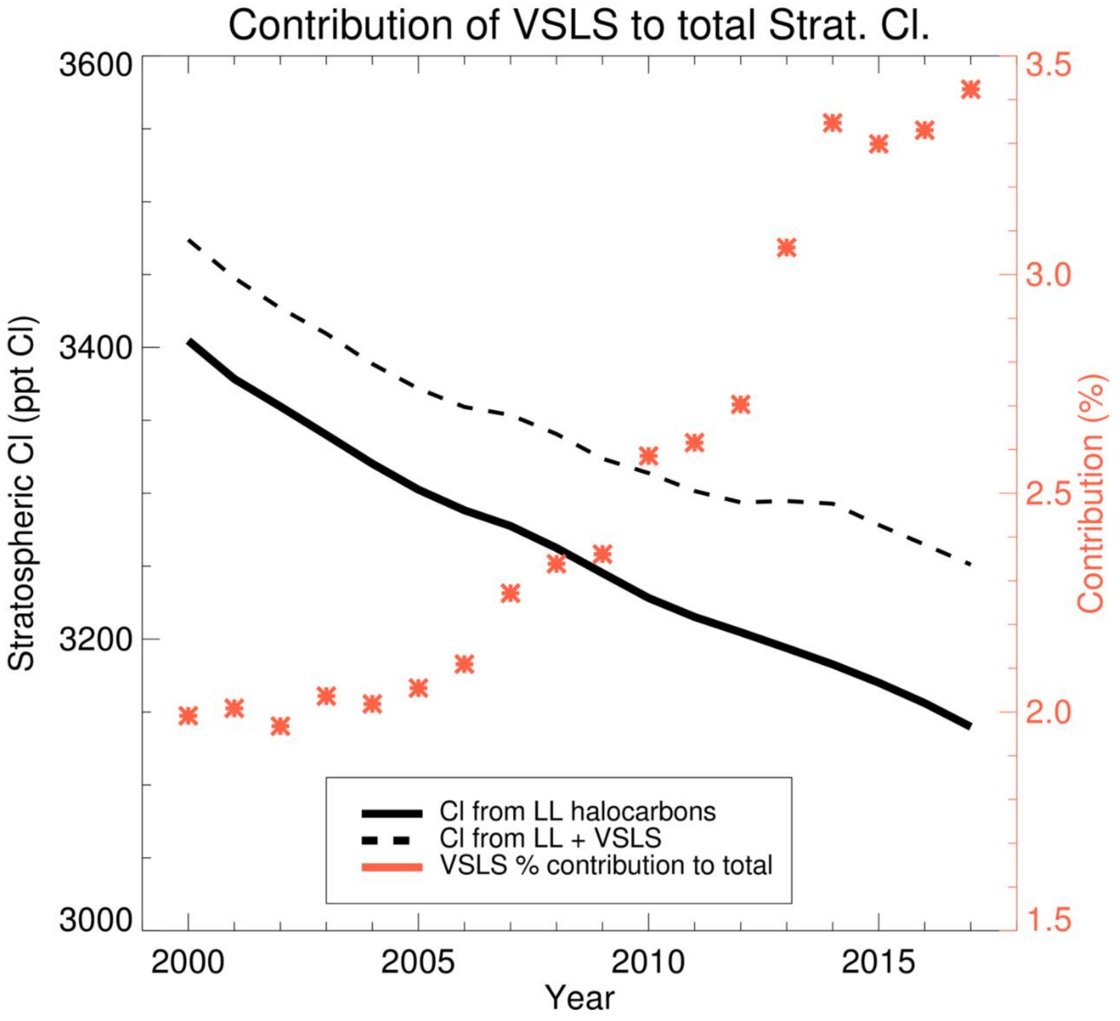

Figure S8. Evolution of total stratospheric chlorine (left y-axis) from long-lived (LL) halocarbons (solid black line) and LL halocarbons + VSLS (dashed line). Also shown is the percentage contribution of VSLS to the total chlorine loading (right y-axis). Model data from BASE simulation is shown. The contribution from long-lived halocarbons is estimated from surface abundances taken from the WMO A1 scenario [WMO, 2014], defined as: 3×CFC11 + 2×CFC12 + 3×CFC113 + 2×CFC114 + CFC115 + 4×CCl_4 + 3×CH_3CCl_3 + HCFC22 + 2×HCFC141b + HCFC142b + Halon1211 + CH_3Cl.

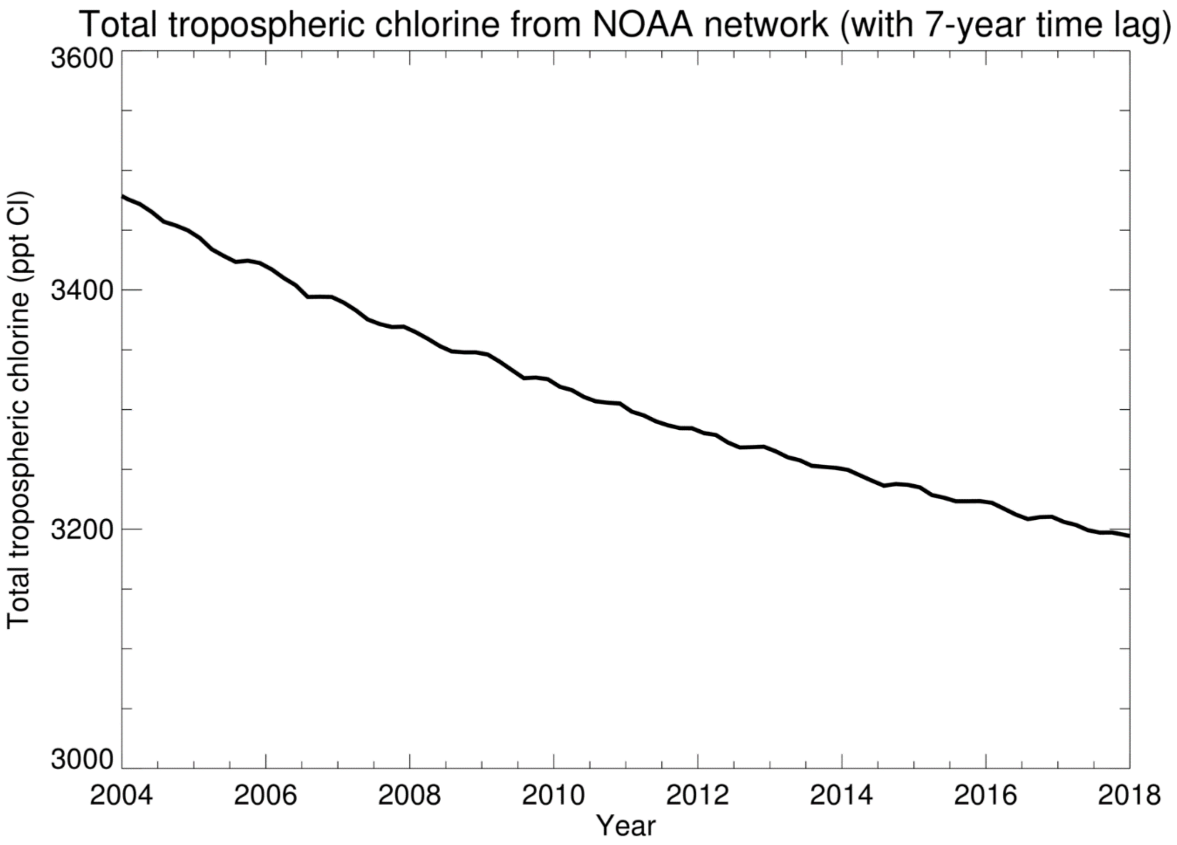

Figure S9. Total tropospheric chlorine (ppt Cl) observed from the NOAA global surface network (with 7-year time lag). Data from: ftp://ftp.cmdl.noaa.gov/hats/Total_Cl_Br/

Table S1. Bimolecular rate constants used in this study

Reaction	**Rate Constant, k** (cm^3 molecules^{-1} s^{-1})	**Reference**
CH_2Cl_2 + OH	1.92×10^{-12}exp (-880/T)	Burkholder et al. (2015)
$CHCl_3$ + OH	2.2×10^{-12}exp (-920/T)	Burkholder et al. (2015)
C_2Cl_4 + OH	4.7×10^{-12}exp (-990/T)	Burkholder et al. (2015)
$C_2H_4Cl_2$ + OH	1.14×10^{-11}exp (-1150/T)	Burkholder et al. (2015)
C_2HCl_3 + OH	8.00×10^{-13}exp (300/T)	Burkholder et al. (2015)
CH_2Cl_2 + Cl	7.4×10^{-12}exp (-910/T)	Burkholder et al. (2015)
$CHCl_3$ + Cl	3.3×10^{-12}exp (-990/T)	Burkholder et al. (2015)
$C_2H_4Cl_2$ + Cl	1.3×10^{-12}	Wallington et al. (1996)
C_2HCl_3 + Cl	7.2×10^{-11}	Catoire et al. (1997)

Table S2. Termolecular rate constants used in this study

**Reaction**	**Rate Constant, k_0** (cm^6 molecules^{-2} s^{-1}) and **k_{inf}** (cm^3 molecules^{-1} s^{-1})	**Reference**
C_2Cl_4 + Cl	k_0 (T) = 1.4×10^{-28} (T/300)$^{-8.5}$<br>k_{inf} (T) = 4.0×10^{-11} (T/300)$^{-1.2}$	Burkholder et al. (2015)

Table S3. Summary of upper stratospheric HCl trends (%/decade) from model simulations and ACE satellite measurements [Bernath and Fernando, 2018]. The trends are for the 2004-2017 period and the 60°S-60°N latitude range (mean at pressures of 1.47 hPa, 1.0 hPa and 0.68 hPa). Errors are ±2 standard error calculated using the expression given in Bernath and Fernando [2018] that accounts for the autocorrelation in the residuals; based on Weatherhead et al. [1998].

**Estimate**	**HCl trend (%/decade)**
Model S-NOVSLS	-6.1 ±0.21
Model S-BASE	-5.2 ±0.32
Model S-EXP3	-5.1 ±0.32
Model S-FIXDYN	-5.0 ±0.31
ACE satellite	-4.8 ±0.69
